# Combined flow cytometry and high-throughput image analysis for the study of essential genes in *Caenorhabditis elegans*

**DOI:** 10.1186/s12915-018-0496-5

**Published:** 2018-03-29

**Authors:** Blanca Hernando-Rodríguez, Annmary Paul Erinjeri, María Jesús Rodríguez-Palero, Val Millar, Sara González-Hernández, María Olmedo, Bettina Schulze, Ralf Baumeister, Manuel J. Muñoz, Peter Askjaer, Marta Artal-Sanz

**Affiliations:** 1 Andalusian Center for Developmental Biology, Consejo Superior de Investigaciones Científicas/Junta de Andalucía/Universidad Pablo de Olavide, Seville, Spain; 2 Department of Molecular Biology and Biochemical Engineering, Universidad Pablo de Olavide, Seville, Spain; 3 GE Healthcare Life Sciences, Maynard Centre, Forest Farm, Whitchurch, Cardiff, UK; 4 Present address: Target Discovery Institute, Nuffield Department of Medicine, University of Oxford, Oxford, UK; 5 Present address: Cell and Developmental Biology Area, Centro Nacional de Investigaciones Cardiovasculares Carlos III (CNIC), Madrid, Spain; 6 Present address: Department of Genetics, University of Seville, Seville, Spain; 7 Centre for Biological Signalling Studies (BIOSS), Laboratory for Bioinformatics and Molecular Genetics, Faculty of Biology, and ZBMZ Center for Biochemistry and Molecular Cell Biology (Faculty of Medicine), Albert-Ludwigs-University of Freiburg, Freiburg, Germany

**Keywords:** *C. elegans*, Essential genes, Worm sorting, Image analysis, High-content, High-throughput, Screens, UPR^mt^, Mitochondria, Prohibitins

## Abstract

**Background:**

Advances in automated image-based microscopy platforms coupled with high-throughput liquid workflows have facilitated the design of large-scale screens utilising multicellular model organisms such as *Caenorhabditis elegans* to identify genetic interactions, therapeutic drugs or disease modifiers. However, the analysis of essential genes has lagged behind because lethal or sterile mutations pose a bottleneck for high-throughput approaches, and a systematic way to analyse genetic interactions of essential genes in multicellular organisms has been lacking.

**Results:**

In *C. elegans*, non-conditional lethal mutations can be maintained in heterozygosity using chromosome balancers, commonly expressing green fluorescent protein (GFP) in the pharynx. However, gene expression or function is typically monitored by the use of fluorescent reporters marked with the same fluorophore, presenting a challenge to sort worm populations of interest, particularly at early larval stages. Here, we develop a sorting strategy capable of selecting homozygous mutants carrying a GFP stress reporter from GFP-balanced animals at the second larval stage. Because sorting is not completely error-free, we develop an automated high-throughput image analysis protocol that identifies and discards animals carrying the chromosome balancer. We demonstrate the experimental usefulness of combining sorting of homozygous lethal mutants and automated image analysis in a functional genomic RNA interference (RNAi) screen for genes that genetically interact with mitochondrial prohibitin (PHB). Lack of PHB results in embryonic lethality, while homozygous PHB deletion mutants develop into sterile adults due to maternal contribution and strongly induce the mitochondrial unfolded protein response (UPR^mt^). In a chromosome-wide RNAi screen for *C. elegans* genes having human orthologues, we uncover both known and new PHB genetic interactors affecting the UPR^mt^ and growth.

**Conclusions:**

The method presented here allows the study of balanced lethal mutations in a high-throughput manner. It can be easily adapted depending on the user’s requirements and should serve as a useful resource for the *C. elegans* community for probing new biological aspects of essential nematode genes as well as the generation of more comprehensive genetic networks.

**Electronic supplementary material:**

The online version of this article (10.1186/s12915-018-0496-5) contains supplementary material, which is available to authorized users.

## Background

Essential genes are critical for organismal development and are often associated with human diseases [1]. However, systematic analysis of essential gene function is being conducted at a slower pace than that of non-essential genes, in particular in multicellular model organisms as compared to yeast [2].

Approximately 30% of the genes in the *Caenorhabditis elegans* genome are essential [3, 4]. To investigate essential genes, multiple approaches can be used that temporarily or partially reduce gene function [5]. Amongst them, temperature-sensitive (ts) alleles are extensively used, where protein function can be disturbed specifically upon temperature shift at any desired time. Although the number of identified ts alleles is rapidly increasing [6–9], not even 10% of the ~ 7000 essential genes in *C. elegans* have a ts allele as of now. An alternative to maintain and propagate lethal mutations is the use of balancer chromosomes. Around 85% of the *C. elegans* genome has been successfully balanced by large genomic rearrangements, and community efforts aim at covering the complete genome [10–12]. Balancers prevent recombination with their homologous chromosomes and, therefore, loss of lethal alleles in a population. Balancers carry alleles that negatively affect reproductive fitness when carried in homozygosity. In addition, an increasing number of chromosome balancers carry fluorescent transgenes, making the identification of homozygous mutant animals easy, as they lack the fluorescent marker [11]. However, such strains are not easy to manage for large-scale analyses, as manual isolation of the homozygous mutant population of interest is far too labour-intensive.

One possible solution for the tedious task of selecting large numbers of homozygous worms could be automated worm sorting using the flow cytometer COPAS (Complex Object Parametric Analyzer and Sorter) Biosort system (“worm sorter”, Union Biometrica, Holliston, MA, USA) [13]. Recently, it has been utilised for sorting balanced mutants at the L3 [14] and L4 [15] larval stages to collect enough biological material for molecular biology techniques. Similarly, COPAS has contributed significantly to high-throughput and high-content analyses [16–18], like isolating homozygous young adults to perform image-based high-content assays to measure germ cell fate reprogramming [19]. The integration of automated worm sorting with microscopy platforms that facilitate automated image acquisition, worm segmentation and data analysis has advanced existing genome-wide screening strategies [20–23]. Moreover, the small size and transparency of the worm coupled with systemic RNA interference (RNAi) methodology has facilitated high-throughput and high-content whole animal screenings using fluorescent protein reporters or dyes, as well as drug screening [24–29]. However, sorting of homozygous mutants from a balanced strain has never been used to study genetic interactions of essential genes.

Here we present an automated sorting and imaging strategy to screen for genetic interactions of essential genes whose loss-of-function alleles can be maintained using fluorescently labelled chromosome balancers. In particular, we optimised a sorting protocol capable of distinguishing homozygous mutants that induce a green fluorescent protein (GFP)-labelled stress reporter from balanced worms expressing GFP in their pharynx. Afterwards, we implemented a segmentation protocol for image analysis that distinguishes eventual green pharynxes that can subsequently be discarded from the analysis using the Developer Toolbox software (GE Healthcare). The protocol includes immediate background subtraction of all segmented worms, and it measures, in addition to fluorescence intensity, a variety of other parameters like area, length, curvature, etc. that can be defined by the user. We re-created the image analysis protocol using CellProfiler [30], a free and open source image analysis software that caters for a variety of assays irrespective of the imaging system used. This protocol is easily adaptable to the user’s needs in terms of different fluorescent markers and image formats and resolutions. Doing so, we complement the features of the CellProfiler “WormToolbox”, a toolbox for high-throughput screening of image-based *C. elegans* phenotypes [23, 31] and make the protocol available to the broader community. We present validation that both protocols produce comparable results.

We provide proof of concept of the sorting and image analysis protocols by carrying out an RNAi screen in the mitochondrial prohibitin deletion mutant *phb-2(tm2998)*. The prohibitin (PHB) complex, a ring-like structure in the inner mitochondrial membrane, is composed of two subunits, PHB-1 and PHB-2 [32]. Loss of either of the subunits leads to the absence of the whole complex, both in unicellular and multicellular eukaryotes [33, 34]. Prohibitins are strongly evolutionarily conserved proteins [35, 36], suggesting an important cellular function. While deletion of PHB does not cause any observable growth phenotype in the unicellular yeast *Saccharomyces cerevisiae* [37], in multicellular organisms such as *C. elegans* [33] and mice [38], where it is ubiquitously expressed, the PHB complex is required for embryonic development. Post-embryonic depletion of the complex by RNAi in *C. elegans* causes severe germline defects [33]. Despite the fact that their exact molecular function is yet to be deciphered [35, 36], PHB complexes have been implicated in several age-related diseases [39, 40] and are involved in mitochondrial morphogenesis and maintenance of mitochondrial membranes by acting as scaffolds [41] or as chaperones that assist with protein folding and degradation [42]. Recently, PHB-2 has been described as being essential for Parkin-mediated mitophagy [43]. Notably, homozygous prohibitin deletion mutants develop into sterile adults that strongly induce the mitochondrial unfolded protein response (UPR^mt^). Upon mitochondrial stress, cells respond by activating dedicated chaperones and proteases of the UPR^mt^ [44–46]. We used quantitative measurements of P*hsp-6::*GFP expression as a readout for the induction of the UPR^mt^, as well as worm size, with the aim of identifying genetic interactors of prohibitins and mechanisms modulating the UPR^mt^. We provide evidence that our methodology can detect both types of interactions.

Our protocol allows the characterisation of genetic interactions of essential genes by applying high-content screening for a variety of phenotypes, including expression of a particular gene of interest (e.g. stress reporters). The combination of nematode sorting along with accurate high-throughput imaging further strengthens *C. elegans* as a powerful organism for the functional genomic analysis of essential genes.

## Results

In the following sections, we demonstrate the usefulness of our sorting and image analysis protocol in identifying genetic interactions of essential genes, when mutants of these can be maintained using fluorescently labelled chromosome balancers. We first describe the phenotypes of mitochondrial prohibitin deletion mutants. We follow by describing in detail the sorting and imaging protocols used to perform systematic RNAi screens and providing experimental evidence for the wide applications of the protocol utilising prohibitin mutants as an example. Finally, we compare the performance of the Developer Toolbox protocol with the protocol based on the free and open source CellProfiler software.

### Characterisation of *C. elegans* mitochondrial prohibitin deletion mutants

**Fig. 1:**
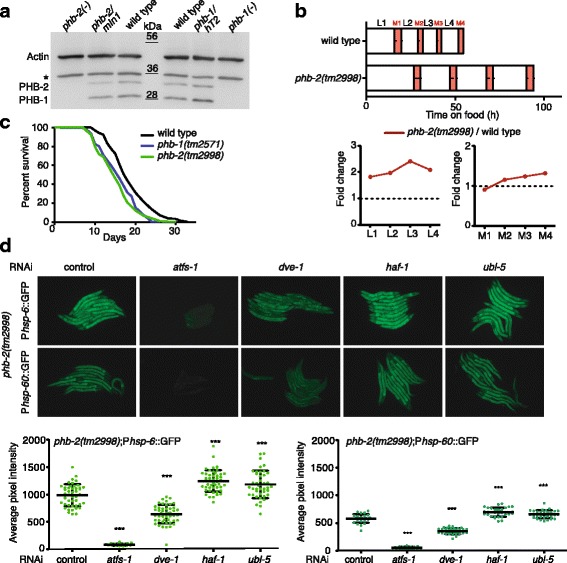
Characterisation of *phb-2(tm2998)* mutants. **a** Western blot showing PHB protein levels. In homozygous *phb-1(tm2571)* and *phb-2(tm2998)* mutants, both proteins, PHB-1 and PHB-2, are undetectable. Actin is used as loading control. *Asterisk* denotes unspecific band. One representative western out of three is shown. **b** Average duration of development for wild-type (*n* = 12) and *phb-2*(*tm2998)* mutants (*n* = 18) at 20 °C, using the LUC::GFP bioluminescent reporter. *Red* represents the molts as inferred by low LUC signal. Error bars represent the standard deviation (SD) of the duration of each interval. The graphs below represent the duration of larval stages (L1–L4) and molts (M1–M4) normalised to wild type. The duration of all larval stages (L1–L4) is significantly different between wild type and *phb-2(tm2998)* mutants (*P* value < 0.001; two-tailed unpaired *t* test). The duration of the M2–M4 molting cycles is significantly different amongst wild type and *phb-2(tm2998)* mutants, with the exception of M1 (*P* value < 0.001; two-tailed unpaired *t* test). A representative experiment of two independent replicas is shown. **c** Lifespans of *phb-1(tm2571)* (mean = 17±1 days, *n* = 215) and *phb-2(tm2998*) (mean = 14.5±0.5 days, *n* = 302) are significantly shorter than that of the wild type (mean = 18 days, *n* = 137) (*P* value < 0.0001, log-rank (Mantel-Cox)). Average of two independent assays is shown. **d** Background-dependent induction of UPR^mt^ reporters in prohibitin deletion mutants. Fluorescent microscopy images of transgenic animals *phb-2(tm2998);*P*hsp-6*::GFP and *phb-2(tm2998);*P*hsp-60*::GFP treated with RNAi against the UPR^mt^ components ATFS-1, DVE-1, HAF-1 and UBL-5. Graphical representation of quantification of P*hsp-6*::GFP (*bottom panel, left*) and P*hsp-60*::GFP (*bottom panel, right*)***.*** The induced UPR^mt^ in prohibitin deletion mutants is suppressed upon depletion of *atfs-1* and *dve-1*, whereas the expression of both UPR^mt^ reporters is further increased upon depletion of *haf-1* and *ubl-5*. *n* > 30 in all conditions. (Mean ± SD; ***P value < 0.001; analysis of variance (ANOVA) test.) One independent replicate out of three is shown

In *C. elegans*, homozygous *phb-1* and *phb-2* deletion mutants produced by heterozygous mothers develop into adults due to maternal contribution but are sterile [47] and need to be maintained as balanced heterozygous strains. Here, the *phb-2(tm2998)* deletion was balanced using an inversion on chromosome II, *mIn1,* which carries an integrated pharyngeal GFP element, while for the *phb-1(tm2571)* deletion, we used a reciprocal translocation between chromosomes I and III*, hT2,* also accompanied by an integrated pharyngeal GFP element. Western blot analysis of *phb-1(tm2571)* and *phb-2(tm2998)* deletion mutants confirmed the absence of the PHB complex (Fig. 1a), as PHB-1 and PHB-2 are interdependent for protein complex formation and stability [33]. Therefore, *phb-1* and *phb-2* mutants show identical phenotypes. We used a method based on bioluminescence [48] to accurately measure the developmental timing of homozygous *phb-2* deletion mutants, which exhibited delayed development relative to wild-type animals. All larval stages lasted approximately twice the time of wild-type animals, and the third larval stage showed a more than twofold increase. However, the duration of the molts was either not affected or mildly increased (Fig. 1b). When assayed for longevity, PHB deletion mutants recapitulated the RNAi phenotypes [47], living for a shorter time than wild-type worms (Fig. 1c).

### PHB deletion induces the mitochondrial unfolded protein response by a non-canonical mechanism

Mitochondrial stress triggers the expression of the conserved mitochondrial chaperones HSP-6 and HSP-60, which have been used to screen for components of the UPR^mt^ signal transduction pathway [44–46, 49]. Nuclear genes encoding UPR^mt^ components required for HSP-6 and HSP-60 expression include the putative mitochondrial inner membrane ATP-binding cassette (ABC) transporter protein HAF-1 that exports the peptides resulting from the cleavage of unfolded proteins to the cytosol [45, 49]. These peptides trigger an unknown signalling cascade that results in the nuclear localisation of the bZIP transcription factor ATFS-1 [49, 50]. Additional transcriptional regulators of the UPR^mt^ are the transcription factor DVE-1 [45] and the ubiquitin-like protein UBL-5, which physically interact [44].

RNAi depletion of either *phb-1* or *phb-2* strongly induces the UPR^mt^ [46, 51, 52]. To monitor the UPR^mt^ in PHB deletion mutants, we incorporated the UPR^mt^ reporters P*hsp-6*::GFP and P*hsp-60*::GFP [46] in *phb-2(tm2998)* mutants. The transcription factors ATFS-1 and DVE-1 appeared to be required for full induction of the UPR^mt^ in *phb-2* mutants (Fig. 1d). However, HAF-1 and UBL-5 were not necessary for the PHB-mediated activation of the UPR^mt^; instead, their depletion further increased the expression of both UPR^mt^ reporters (Fig. 1d). We confirmed this observation using *haf-1(ok705)* deletion mutants, where RNAi depletion of either *phb-1* or *phb-2* increased the UPR^mt^ significantly more than in otherwise wild-type animals (Additional file 1: Figure S1a). Similar to *haf-1(RNAi)* treatment, *haf-1(ok705)* deletion enhanced the P*hsp-6::*GFP expression in *phb-2(tm2998)* deletion mutants (Additional file 1: Figure S1b). This suggests that HAF-1 not only is dispensable for signalling the UPR^mt^ upon prohibitin depletion, but that blocking peptide transport through HAF-1 increases mitochondrial stress when depleting either subunit of the PHB complex. Similarly, we observed enhanced expression of P*hsp-6::*GFP in *haf-1(ok705*) deletion mutants upon RNAi against the mitochondrial AAA protease *spg-7* (Additional file 1: Figure S1c), which cooperates with the PHB complex in mitochondrial quality control [53]. This observation is contrary to previously published data [49], but is in agreement with a previous report showing that *haf-1* is not required for induction of the UPR^mt^ caused by RNAi knockdown of *phb-2* [52].

Together, these data indicate that an alternative mitochondria-to-nucleus signalling mechanism might exist. We therefore developed an automated method to screen for PHB genetic interactors and regulators of the UPR^mt^. Our method consists of combined automated worm sorting and high-content image analysis that can be adapted and applied to any strain carrying a fluorescently labelled balancer.

### Sorting homozygous prohibitin deletion mutants

**Fig. 2:**
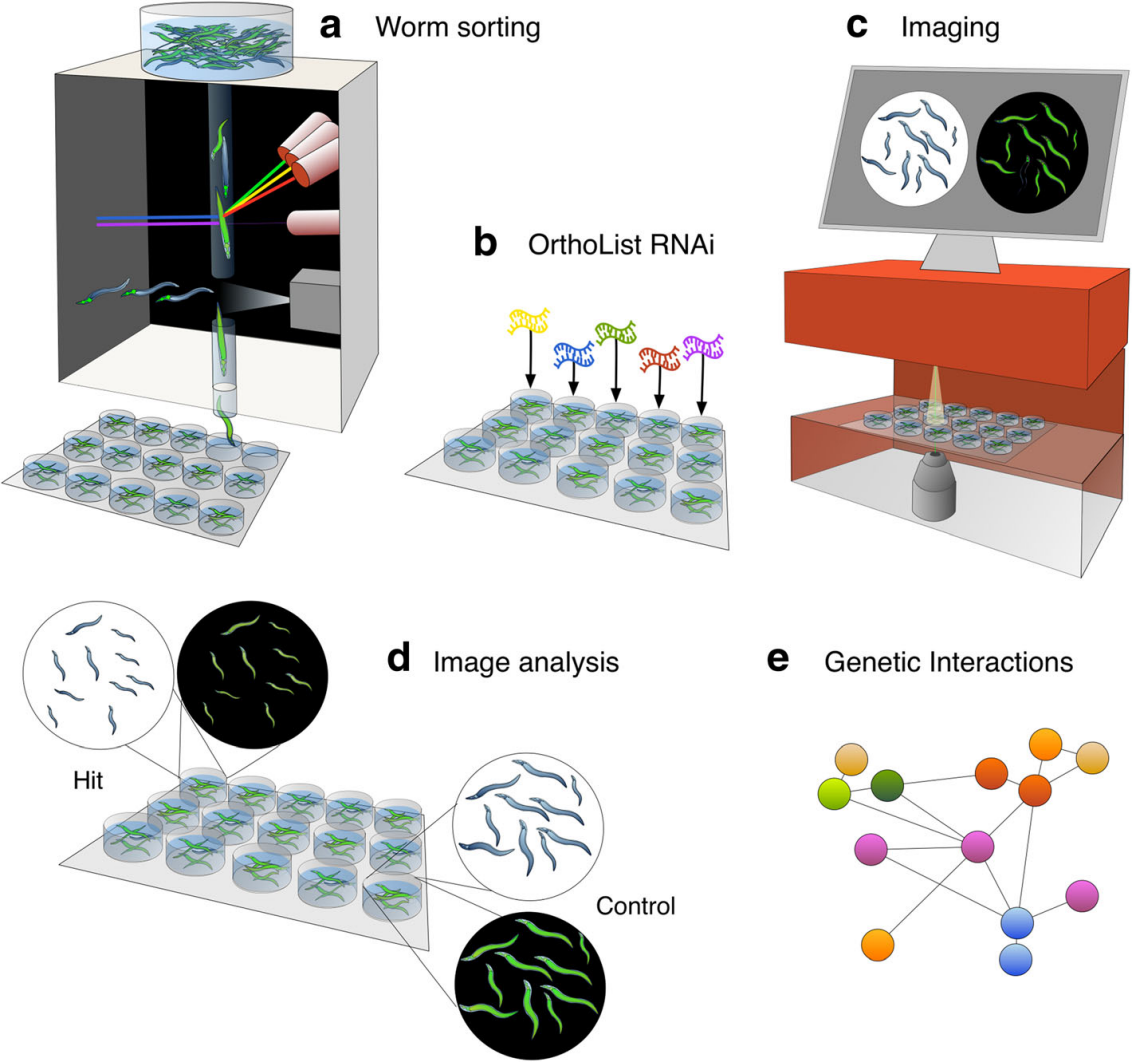
Overview of the screening strategy for the study of essential genes. **a** Homozygous *phb-2(tm2998);*P*hsp6::*GFP mutants are sorted at L2 stage from a mixed population of balanced heterozygous *phb-2(tm2998)/mIn1* animals into multiwell plates using the COPAS Biosort “worm sorting”. **b** Next, bacteria containing the OrthoList RNAi sublibrary are added to the wells and worms are incubated at 20 °C. **c** When worms reach the desired stage, they are imaged in brightfield and fluorescent channels using an automated microscope, IN Cell Analyzer (GE Healthcare). **d** Employing a user-defined image segmentation protocol, hits can be defined based on different measurements like reporter expression or size of the worms in comparison with the control. **e** Finally, hits can be analysed by building genetic networks based on predicted and described protein interactions in different organisms

**Fig. 3:**
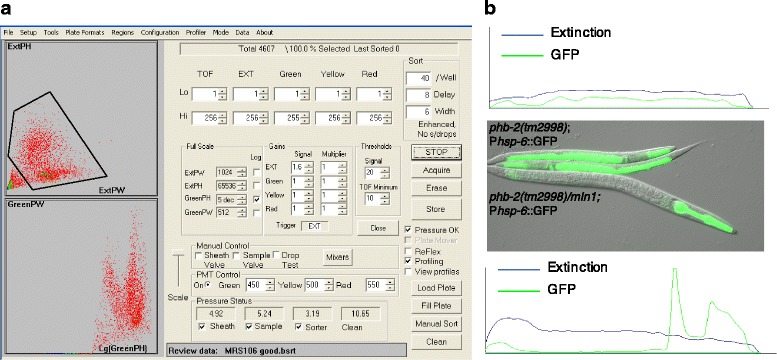
Worm sorting settings based on gating region and Profiler feature. **a** COPAS Biosort conditions optimised for the sorting of homozygous *phb-2(tm2998);*P*hsp-6::*GFP at the L2 stage. The *upper panel* reflects the gating region based on the extinction peak height (*ExtPH*) and the extinction peak width (*ExtPW*) selecting the worm population. The *lower panel* shows the worm distribution based on green parameters (green peak height (*green PH*) and green peak width (*green PW*)). **b** Utilising the Profiler II software to distinguish between *phb-2(tm2998)/mIn1;*P*hsp-6::*GFP and *phb-2(tm2998);*P*hsp-6::*GFP larvae using green parameters. The *phb-2(tm2998)/mIn1;*P*hsp-6::*GFP worms show two peaks in the green profile corresponding to the two lobes of the pharynx, have a green PH above 10,000 and are excluded (*bottom panel*). The *phb-2(tm2998);*P*hsp-6::*GFP larvae show a profile without pronounced peaks, with a green PH ranging from 700 to 10,000 and green PW above 120, and are accepted and sorted (*top panel*). The picture includes a balanced heterozygous *phb-2(tm2998)/mIn1;*P*hsp-6::*GFP expressing P*myo-2*::GFP in the pharynx and two *phb-2(tm2998);*P*hsp-6::*GFP homozygous deletion mutants with induced P*hsp-6::*GFP expression imaged at the moment of sorting, approximately 48 h after synchronisation

We used COPAS to sort homozygous *phb-2(tm2998)* deletion mutants from a mixed population of *phb-2(tm2998)/mIn1* balanced animals carrying the UPR^mt^ stress reporter P*hsp-6*::GFP (Fig. 2). We sorted homozygous *phb-2(tm2998)* deletion mutants at the second larval (L2) stage, to allow RNAi treatment during early development. This is more challenging than what has been accomplished earlier, as we attempted to sort relatively small homozygous *phb-2(tm2998)* worms expressing P*hsp*-6::GFP all along their body from the population carrying the balancer *mIn1* and, hence, expressing a pharyngeal GFP element but not the reporter P*hsp-6*::GFP (Fig. 3b).

The COPAS instrument measures the optical density of the object (extinction), the size of the object and also the fluorescence intensity in three channels: green, yellow and red (Fig. 3a). Based on these parameters, the user can define criteria for sorting and dispensing the population of interest into microtitre plates. In order to make the sorting more accurate, the standard COPAS system is implemented with the Profiler II software. Instead of making a single integrated measurement of a signal, the Profiler gives a list of successive point measurements along the object passing through the flow cell and builds a fluorescence profile. Based on these measurements, it can detect fluorescence intensity peaks along the length of the object (Fig. 3b).

In our case, in order to remove small particles and possible debris and select specifically worms, we defined a gating region based on the extinction peak height (ExtPH) and the extinction peak width (ExtPW) (Fig. 3a). Next, we used the green channel (500–520 nm wavelength) to select animals with green body from the rest of the population. As the green signal coming from the pharynx is more intense than the signal coming from the stress reporter, we accepted a signal range between 700 and 10,000. These numbers refer to the highest value measured along the object. Nevertheless, in some cases, the signal coming from the pharynx is lower; thus, we added a width criterion. To measure the width, the program integrates the widths of all the areas of the profile that exceed the value set up as the lower limit of peak height (700). We accepted widths above 120, excluding signals from the pharynx, which do not have the same extent as the body of the worm. All these numbers can be adjusted depending on the user requirements.

Once animals were sorted into 96-well plates, bacteria producing double-stranded RNA (dsRNA), prepared in parallel (see Additional file 2: Table S1 and Additional file 3), were added to the worms. In order to streamline the identification of relevant PHB interactors for human health, we assembled the OrthoList RNAi sublibrary, maintaining the original name of the published compendium of *C. elegans* genes having human orthologues [54].

### Functional genomic analysis of chromosome I using the OrthoList RNAi library

*C. elegans* is an important invertebrate model for elucidating the mechanisms of conserved pathways relevant to human biology and disease. In *C. elegans*, RNAi can be applied by feeding worms bacteria expressing dsRNA for individual genes [55], which results in the generation of “feeding libraries” covering most of the predicted protein-coding genes in the genome [56, 57]. Of the ~ 20,000 predicted protein-coding genes in *C. elegans* [58], 7663 genes have been listed in the “OrthoList” [54], a compilation of *C. elegans* genes sharing a human orthologue. Of those, about 80% (6329 clones) are present in the Ahringer RNAi feeding library [56]. The generated “OrthoList RNAi sublibrary” contains a total of 6315 RNAi clones, since 14 clones from the Ahringer RNAi library did not grow during the preparation of the sublibrary. The 6315 bacterial clones correspond to 6179 different genes and are organised into 72 96-well plates, leaving empty the last column of the plates for the pertinent controls.

As a quality control for the OrthoList RNAi library, we sequenced 144 widespread clones, of which 131 were identified correctly, corresponding to 91% of the bacterial clones being reliable. In 2011, Qu et al. performed an evaluation of the Ahringer *C. elegans* library [59], carrying out a bioinformatics analysis, and resolved that 98.3% of the clones are trustworthy, even though 17.5% of the clones needed to be re-annotated. Taking into account these numbers, we concluded that the OrthoList RNAi sublibrary that we have generated is a high-quality tool.

Here, we present a functional genomic analysis of chromosome I using the OrthoList RNAi library. Chromosome I has 1207 orthologous genes annotated, which correspond to 14 96-well plates. RNAi was initiated at the second larval stage, and the animals were imaged 2 days later, at the young adult stage (Fig. 1b).

### High-throughput image acquisition and analysis

**Fig. 4:**
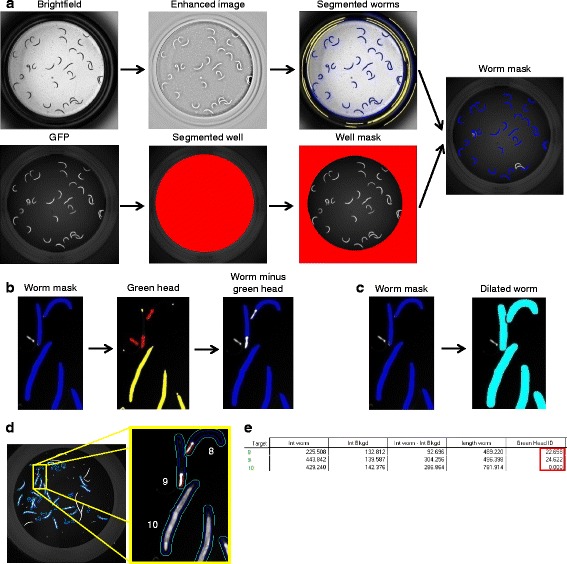
Outline of the GFP image segmentation protocol for balanced mutants. **a** Representative images acquired in brightfield and green channels. First, the well is segmented based on intensity in the green channel, then the image is inverted (*bottom*). The brightfield image is subjected to pre-processing to enhance the contrast for ease of segmentation. The segmented worms are tagged in *blue* and excluded objects are tagged in *yellow* (*top*). Exclusion criteria are specified in Additional file 4. The user can modulate these morphological parameters per requirement. By subtracting the well mask from the segmented worms, we obtain the worm mask. **b** Identification of heterozygous animals (*Green head* ID). Once worm mask is defined, green heads are segmented and tagged in *red*, excluded objects are tagged in *yellow*. Exclusion is based on area and intensity levels. The user can modulate these morphological parameters per requirement. The next step subtracts the identified green heads from the worm bodies. As a result, segmented worms appear *blue* without the green heads (*Worm minus green head*). **c** Background subtraction. Segmented worms are dilated in order to facilitate measurement of the intensity of the immediate background. **d** Target linking in order to compose one final target. Worm mask, dilated worm and worm minus green head targets are linked together. The final target shows the three measurement regions: the worms in *blue*, the immediate background in *cyan* and the green head in *red*. **e** Identified targets with the corresponding measurements. Intensity of the worm (*Int worm*), intensity of the immediate background (*Int Bkgd*), background subtraction (*Int worm - Int Bkgd*), worm length and identification of green head (*Green Head ID*). Targets 8 and 9 correspond to heterozygous worms with a green head ID greater than 0. Additional measures like area, major axis length, *X*/*Y* position and form factor can be added per user requirement

After RNAi treatment, once worms reached the desired stage, we anesthetised the animals and washed off the bacteria from the microtitre plates before imaging. Whole-well brightfield and green fluorescence images were taken sequentially using the IN Cell Analyzer 2000, an automated microscope designed for cell-based high-content screening that we adapted for worm imaging using a 2× objective (Fig. 4a). Image analysis was performed with a user-defined protocol within the Developer Toolbox software (version 1.9.2) (GE Healthcare), accompanying the IN Cell Analyzer 2000 that enables direct upload and analysis of image stacks. In this protocol, we defined four individual targets: a well edge (used to subtract out any well debris), a worm, a dilated worm (used to calculate intensity of the background adjacent to the worm) and a worm with a green head (so these can be segregated from the dataset downstream of image analysis). In order to obtain all the required measurement regions in one target, individual targets were linked together. For two targets to be linked, there must be at least one pixel overlap to achieve the linkage. Each target was created stepwise. First, a pre-process was applied if required, for example to enhance image contrast prior to segmentation. Second, the target was segmented in order to generate a mask, and last, a sequence of post-processing operations was used to clean up the mask. Once the targets were created, optimised and linked together, measurements such as size, shape or intensity in the different channels were acquired on a per-worm basis.

In more detail, we first segmented the well mask using the autofluorescence of the well edge in the green channel, and after post-processing refinement steps, we inverted the resultant segmentation mask to give the well edge (Fig. 4a, bottom). Next, we segmented the worms using the brightfield image; again, we applied a pre-process to create an enhanced image from which we segmented the worms (Fig. 4a, top). After post-processing refinement of the segmented worms, we subtracted the well mask to remove well artefacts. Finally, in order to remove unwanted objects such as long fibres, air bubbles or overlapping worms, we applied acceptance criteria based on morphological parameters and created the worm mask (Fig. 4a). Even though the sorting protocol was largely efficient, some heterozygous worms can be present in the well. Due to the longer developmental time of *phb-2(tm2998)* homozygous mutants (Fig. 1b), the heterozygous worms lay progeny before the mutants reach the young adult stage, and several larvae with green pharynx can be found. We implemented the segmentation protocol by adding a step that identifies worms with a green pharynx. Once the worms were well delineated, we segmented green heads in the green channel image and used acceptance criteria based on area and intensity to better define them (Fig. 4b). In order to measure the intensity of the immediate background of each worm, the worm mask was dilated, creating a dilated worm (Fig. 4c). Eventually, we linked targets to get all regions together. The resultant target has all three measurement regions: a worm mask, a dilated worm mask and a worm minus green head mask (Fig. 4d). We identified worms with a green head by the green head ID value (Additional file 4); a value greater than 0 indicates that a green head is present. A comprehensive list of measures, morphology and intensity-based, for each worm was collected (Fig. 4e). A breakdown of the protocol described here is provided as supplementary information (Additional file 4).

### Screen validation: PHB interactors affecting the UPR^mt^

**Fig. 5:**
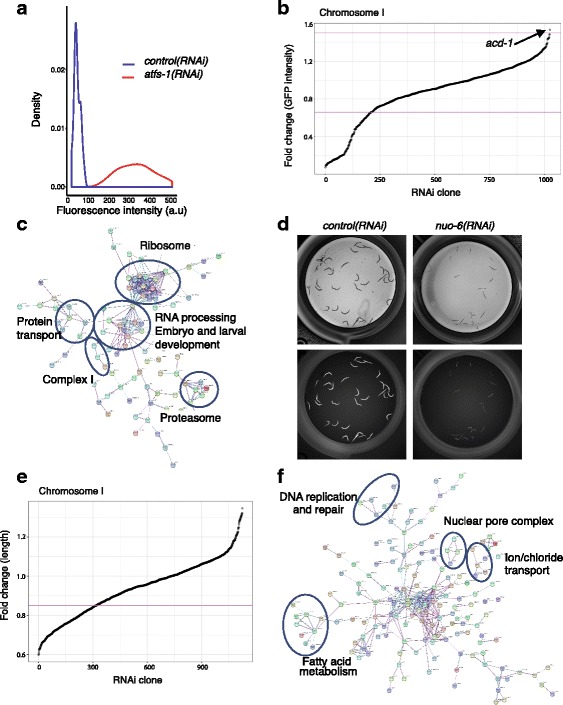
Analysis of the screening data. **a** Density plot (frequency distribution) of the negative controls (control(RNAi)) and positive controls *(atfs-1(RNAi))* based on the GFP intensities. **b** Fold change (FC) of GFP intensity of the 1207 tested RNAi clones against the ordered index. Genes with a *P* value < 0.001 and FC < 0.66 or FC > 1.5 are considered as candidates. Depletion of 208 RNAi clones downregulates P*hsp-6*::GFP signal, whereas only one candidate triggers a further induction of the reporter. **c** Interaction network of the 208 genes whose depletion reduces the UPR^mt^. Networks are built using STRING [76], based on predicted and described interactions in different organisms. *Nodes* are proteins, and the *edges* represent the associations between nodes. *Clusters* show genes involved in processes previously described to be involved in the regulation of the mitochondrial stress response such as ribosome, proteasome, RNA processing, protein transport and complex I of the mitochondrial electron transport chain. **d** Brightfield and green images of control(RNAi) and *nuo-6(RNAi).* Depletion of *nuo-6* triggers a developmental delay in addition to the reduction in the UPR^mt^ reporter expression. **e** FC of worm length of the 1207 tested RNAi clones against the ordered index. Genes with an FC < 0.85 are considered as clones affecting development. Depletion of 303 genes reduces size of the worms. **f** Interaction network of the 303 genes whose depletion reduces size of the worms. Networks are built using STRING [76], based on predicted and described interactions in different organisms. *Nodes* are proteins; *edges* represent the associations between nodes. *Clusters* show genes involved in DNA replication and repair, fatty acid metabolism, ion channels and nuclear pore complex

In order to evaluate changes in GFP expression, an analysis pipeline consisting of several filtering steps, quality control and statistical test was computerised (see Methods section). As shown in Fig. 5a, the negative (empty vector) and the positive *(atfs-1(RNAi))* controls were clearly separable. Our RNAi screen was based on *C. elegans* genes annotated to chromosome I sharing orthologues with humans annotated to chromosome I. We found 208 genes to be necessary for the induction of the mitochondrial stress response, while only the inactivation of one gene, *acd-1,* induced the reporter signal to a greater extent (Fig. 5b). *acd-1* is an orthologue of members of the human SCNN (Sodium channels epithelial) family, which is involved in response to acidic pH and predicted to have sodium channel activity, based on protein domain information.

Under our conditions, we identified 45.5% of previously described genes required for the activation of the UPR^mt^ [44, 45, 49, 60, 61] (Additional file 5). Moreover, based on functional annotation clusters, we found new genes never described to be involved in the regulation of the UPR^mt^ but belonging to pathways already established to suppress the mitochondrial stress response (Fig. 5c and Table 1). We identified a large group of genes encoding proteins of the two ribosomal subunits, genes encoding proteins associated with protein transport including nuclear importins, genes encoding proteasomal subunits and genes involved in mRNA processing. In addition, we encountered a large number of genes involved in embryonic and larval development. Interestingly, as shown in Fig. 5c, we detected as well three genes encoding for subunits of the mitochondrial NADH dehydrogenase complex (Complex I), *nuo-2*, *nuo-6* and *D2030.4*. Looking closely at these worms, we detected that they showed a developmental defect (Fig. 5d).

**Table 1:** Candidates reducing the UPR^mt^ in *phb-2* deletion mutants and annotated to pathways previously involved in UPR^mt^ regulation

Ribonucleoproteins	
*rps-15*	40S ribosomal protein S15
*rps-19*	40S ribosomal protein S19
*rla-0*	60S acidic ribosomal protein P0
*C37A2.7*	60S acidic ribosomal protein P2
*rpl-1*	60S ribosomal protein L10a
*rpl-13*	60S ribosomal protein L13
*rpl-17*	60S ribosomal protein L17
*rpl-7*	60S ribosomal protein L7
*mrpl-24*	Probable 39S ribosomal protein L24, mitochondrial
*snr-7*	Probable small nuclear ribonucleoprotein G
*snr-2*	Probable small nuclear ribonucleoprotein-associated protein
*rpl-14*	Ribosomal protein, large subunit
*rpl-30*	Ribosomal protein, large subunit
*rps-10*	Ribosomal protein, small subunit
*rps-20*	Ribosomal protein, small subunit
Protein transport	
*apg-1*	AdaPtin, gamma chain (clathrin-associated complex 1)
*sec-8*	Exocyst complex component 4
*ran-4*	Probable nuclear transport factor 2
*bbs-9*	Protein pthb1 homolog
*rab-11.1*	Ras-related protein rab-11.1
*nsf-1*	Vesicle-fusing ATPase
*apb-3*	Hypothetical protein
*xpo-2/imb-5*	Importin beta-like protein
*imb-3*	Importin beta-like protein
Proteasome	
*rpn-10*	26S proteasome non-ATPase regulatory subunit 4
*pas-3*	Proteasome subunit alpha type-4
*pas-4*	Proteasome subunit alpha type-7
*pbs-5*	Proteasome subunit beta type
*rpt-5*	Proteasome regulatory particle, ATPase-like
mRNA processing/splicing	
*snr-7*	Probable small nuclear ribonucleoprotein G
*snr-2*	Probable small nuclear ribonucleoprotein-associated protein B
*cel-1*	mRNA capping enzyme-like

### Screen validation: PHB interactors affecting development

In addition to the quantification of P*hsp-6*::GFP expression, the image analysis allows us to obtain many other measurements. In order to identify genetic interactors of PHB-2, we analysed the size of the worms in the same manner as described for the *hsp-6* reporter expression but filtering only based on the absence of green pharynx (green head ID = 0), keeping all worms irrespective of their size. In this case, we set up a threshold of fold change (FC) < 0.85 to obtain the clones affecting size. We found 303 RNAi clones whose depletion led to smaller size, probably due to developmental delays (Fig. 5e). The Ahringer laboratory has performed multiple RNAi screens and assigned biological functions to many genes [56, 62]. In addition, Ahringer and colleagues described many phenotypes, such as embryonic lethal or developmental delay, associated with RNAi depletion of individual genes in wild-type worms. Thirty-one percent of described RNAi clones showing a developmental delay in wild-type animals also affected development of *phb-2* mutants. We expected to have a developmental phenotype in *phb-2* mutants upon depletion of genes causing this phenotype in wild-type worms, although it should be noted that the screening conditions are not the same. Fraser and Kamath [56, 62] subjected worms to RNAi from eggs, whereas we did it from the second larval stage. As expected, amongst the genes affecting development we found genes encoding ribosomal subunits, proteasome subunits and genes involved in protein transport. Moreover, we identified many genes implicated in fatty acid metabolism, such as *lbp-5, acox-1.3 (F08A8.3), F10G8.9, acdh-3, acdh-4* and *ech-1.2 (T08B2.7)* (Additional file 6). By analysing protein-protein interactions (Fig. 5f), we described a cluster of genes encoding for nuclear pore complex proteins (*npp-2*, *npp-4*, *npp-6* and *npp-7*), as well as a cluster of genes involved in DNA replication/repair (*cdt-1*, *crn-1*, *msh-6* and *rpa-4*) and meiotic spindle organisation (*aspm-1*) and chromosome segregation (*icp-1*).

### Additional features of the imaging protocol: analysis of prohibitin deletion mutants stained using the fluorescent dye Nile Red

**Fig. 6:**
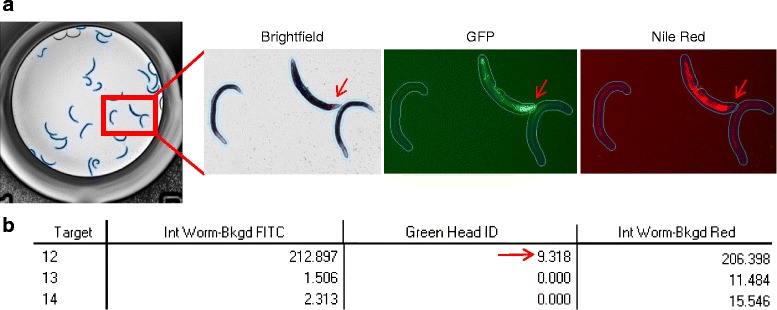
Combined green/red image analysis protocol. **a** Images acquired in brightfield, green (GFP) and red (Nile Red) channels with the IN Cell Analyzer 2000 and processed using Developer Toolbox software (GE Healthcare). A balanced heterozygous animal expressing pharyngeal GFP has been identified (*red arrow*) within a population of homozygous *phb-2(tm2998)* mutants. The heterozygous animal exhibits increased Nile Red staining in comparison to the homozygous *phb-2(tm2998)* mutants. **b** Segmentation analysis output of the green/red image analysis for balanced mutants. Worm intensity subtracting the background intensity in the green image (*Int Worm-Bkgd FITC*), identification of green head (*Green Head ID*) and worm intensity subtracting the background intensity in the red image (*Int Worm-Bkgd Red*) are depicted in the analysis output. Target 12, with a green head ID greater than 0, corresponds to the heterozygous worm. Additional measures like area, major axis length, *X*/*Y* position and form factor can be added per user requirement

In addition to inducing a strong mitochondrial stress response, prohibitin depletion results in significant alterations in Nile Red fluorescence [47]. In order to assess this phenotype, we developed an image acquisition and segmentation protocol that incorporates intensity measurements from an additional channel into the above-described image acquisition and segmentation protocols for balanced mutants (Fig. 6). Nile Red emissions are efficiently captured by the Cy3 filter, referred to hereupon as the red channel. For this protocol, sequential images of whole wells in brightfield, green and red channels were acquired with the IN Cell Analyzer 2000. Image analysis was facilitated by a slightly modified version of the user-defined segmentation protocol for GFP images. This modified protocol follows the same initial steps of well segmentation in the green channel image and segmentation of worms in the brightfield image, followed by subtraction of the well edge, detection and identification of heterozygous worms with pharyngeal GFP and, finally, dilation of the worm for measurement of the immediate background (Fig. 4a–c and Fig. 6a). Acceptance criteria based on morphological parameters were applied to remove unwanted objects. Target linking was done to achieve a final target comprising the final worm mask, the dilated worm and the worm minus green head linked together (Additional file 7: Figure S2). Defined measures were collected for each worm covering morphology and intensity-based measurements from both the green and the red channels (Fig. 6b). A breakdown of the protocol is provided in Additional file 8.

### Open sourcing of the segmentation protocol through CellProfiler

The segmentation protocols presented above are only compatible with the Developer Toolbox software; hence, usage would be limited to researchers with access to the GE platform. To circumvent this, we implemented the protocol in the free and open source CellProfiler software [23, 30], providing an analysis pipeline identifying green head worms, and measuring intensities in the green and red channels.

**Fig. 7:**
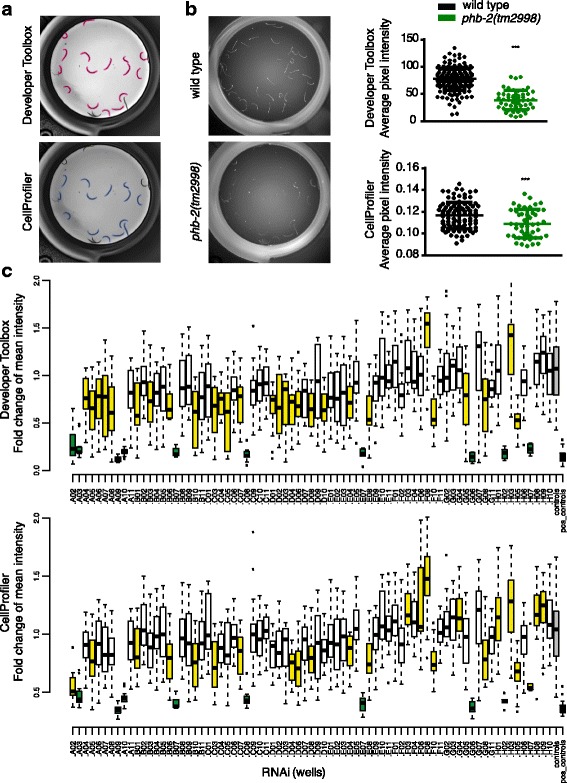
Open source segmentation protocol (CellProfiler). **a** Comparison of the image segmentation output for *phb-2(tm2998)* mutants generated from Developer Toolbox (GE Healthcare) versus CellProfiler. **b** Nile Red staining of *phb-2(tm2998)* mutants versus wild-type animals. Representative images taken with the IN Cell Analyzer and graphical representation of data coming from the different segmentation protocols. As previously shown [47], depletion of *phb-2* reduces Nile Red staining using both of the protocols. (Mean±SD; ***P value < 0.0001 (Developer Toolbox) and ***P value = 0.0008 (CellProfiler); two-tailed unpaired *t* test; *n* > 45 for both conditions, combination of 3–5 different wells.) **c** Comparison of the UPR^mt^ reporter signal after image analysis from Developer Toolbox (GE Heathcare) versus CellProfiler. The box plots represent fold change (FC) mean intensities of the RNAi clones from a randomly chosen 96-well plate from the UPR^mt^ RNAi screen. RNAi clones with a *P* value < 0.001 appear in *yellow* and candidates with a *P* value < 0.001 and an FC < 0.5 appear in *dark green*. Control wells (control RNAi) appear in *grey* and positive control (*atfs-1(RNAi)*) in *black*. By comparing both box plots, we see that results from the different segmentation protocol are highly similar

We compared worm segmentation outputs resulting from the Developer Toolbox and CellProfiler and found them comparable (Fig. 7a). Intensity measures obtained from the Developer Toolbox and CellProfiler in the green and red fluorescence channels were also compared. For the red channel, we compared wild-type animals and *phb-2(tm2998)* mutants after Nile Red staining. Depletion of PHB by RNAi in wild-type animals results in low Nile Red staining [47]. Similarly, we observed lower Nile Red staining in *phb-2* mutants irrespective of the software used for segmentation and quantification (Fig. 7b). For the green channel protocol, we randomly selected one plate from the RNAi screen performed using *phb-2(tm2998);*P*hsp-6*::GFP animals and re-analysed the images using the CellProfiler software. After data processing, the candidates resulting from both analyses were highly similar (Fig. 7c), thus validating the method.

The image analysis protocol generated through the CellProfiler software is provided as Additional file 9, and is easily adaptable to the user’s needs in terms of different fluorescent markers, image formats and image resolutions.

## Discussion

*C. elegans* is an excellent multicellular model for genome-wide studies. Its short and well-defined life cycle, its inexpensive and easy maintenance and its completely sequenced genome, sharing a high degree of sequence conservation with humans, make the nematode a suitable platform for high-throughput and high-content screens. Despite all the advantages, large-scale studies in *C. elegans* involving essential genes are still not well developed. Temperature-sensitive alleles allow the temporal suppression of gene function; however, very few essential genes count with a ts allele [5, 6]. Another possibility is the use of chromosome balancers, which already cover 85% of the genome, and big efforts are being carried out to have fluorescently labelled balancers for all *C. elegans* essential genes via CRISPR ([12] and *Caenorhabditis* Genetics Center (CGC), personal communication).

Here we present a successful strategy for automated whole animal image-based RNAi screening that can be applied to essential genes when carrying a fluorescently labelled balancer and a gene expression reporter. Developing this automated pipeline was demanding due to two major steps in the workflow. First, one must sort homozygous mutant animals expressing a GFP-based stress reporter from a GFP-marked balanced population at an early larval stage (L2), and second, the process requires a robust automated imaging and segmentation protocol using a microscopy platform not previously utilised for *C. elegans*. Most gene expression reporters in *C. elegans* consist of a given promoter fused to GFP (e.g. the *C. elegans* promoterome [63] or numerous metabolic and stress reporters [44, 64]). By exploiting the Profiler II software feature of the worm sorter, we successfully optimised a protocol for sorting homozygous L2 larvae with an efficiency greater than 95%. We first attempted to sort L1 larvae in order to expose the animals to RNAi from the beginning of larval development. However, the pharynx of the animals occupies approximately one third of the body length at the L1 stage, complicating the profiling. Starting the RNAi treatment at the L2 stage was sufficient to uncover relevant genetic interactions. Nonetheless, interactions that take place during the L1 stage might have been overlooked. COPAS is a convenient approach for high-throughput analysis involving balanced strains, as it facilitates a task that manually would be impracticable. For balanced strains in the absence of a gene reporter or when the reporter carries a different fluorophore, the sorting of worms might not require the use of the Profiler.

High-throughput imaging strategies specific to *C. elegans* have been developed using various microscopy platforms [20, 21, 23, 65]. Like others, the IN Cell Analyzer (GE Healthcare) combines brightfield- and fluorescence-based imaging. We optimised image acquisition to include an entire well of a 96-well plate in a single image, ensuring even brightfield illumination by sealing the 96-well plate with a transparent seal. The optimised autofocus and scanning times of the IN Cell Analyzer ensure image acquisition of an entire 96-well plate in two channels in less than 10 min and in three channels in under 15 min. We present here the first automated segmentation protocol for balanced strains in *C. elegans*. We built a user-defined, user-friendly protocol using the Developer Toolbox (GE Healthcare) software. Our segmentation protocol allows intensity-based measurements apart from other measures defined by the user like area, length, curvature, etc., and it can be used for any strain without the need of transgenesis, as segmentation is done in the brightfield image. One novelty of this segmentation protocol is the measurement of the background surrounding each target. By dilating the targeted worm, we can easily measure and subtract the intensity of the immediate background. This is very appropriate in the cases of dyes that stain plastic and can cause different background intensities depending on the area of the well. Another strong point of our image analysis protocol is the successful identification of worms carrying a pharyngeal GFP element. As COPAS sorting is not 100% efficient, heterozygous worms will develop into fertile adults and lay progeny in the wells; hence, the need of distinguishing worms carrying the GFP-balancer from homozygous mutants.

The COPAS can serve as an alternative to microscopy-based measurements, as it can measure length, optical density and fluorescence emission of single worms [18, 66]. However, image-based microscopy platforms have several advantages: they are faster and images are stored, making them accessible for re-analysis. Also, a much smaller number of animals can be used for image-based assays as compared to the COPAS, thus allowing for exploration of a large number of different treatments in parallel, such as RNAi or drug screens. Another advantage of image-based screens is that multiple outputs can be examined from the image, e.g. fluorescent intensities in different channels, size and shape measurements. This helped us to define developmental phenotypes and could be used as well to detect other phenotypes such as sterility by classifying as progeny the small worms (worm length measurement), or to distinguish between thin and fat worms (worm width measurement). We have translated the image analysis protocol for CellProfiler [30], a free and open source image analysis software, making the protocol available to the scientific community. In doing so, we broaden the features of the CellProfiler “WormToolbox” for high-throughput screening of image-based *C. elegans* phenotypes [23, 31]. We provide validation that both protocols produce comparable results. Although the segmentation output is not exactly the same in terms of number of worms segmented, the level of stringency in the segmentation criteria can be easily modulated with both protocols.

Prior to the realisation of the screen, different optimisation steps were needed. Different numbers of worms per well and amounts of bacteria were tested. The final solution is a compromise between having enough worms per well without much overlap, as worms that cross each other are discarded from the analysis. The amount of bacteria needs to be sufficient to avoid starvation, but not too much to prevent anoxic conditions. The speed of shaking during the RNAi treatment has been optimised to prevent the formation of a thick layer of bacteria. Finally, it is worth highlighting the convenience of performing the screen using the same conditions (same batch of plates, same incubator, etc.) to reduce variability.

PHB genes are highly conserved from yeast to mammals. Therefore, to test our automated screening strategy, we performed a chromosome I RNAi screen utilising the OrthoList RNAi library looking for PHB genetic interactors and regulators of the UPR^mt^. The OrthoList RNAi library that we have created will be of considerable advantage for *C. elegans* researchers to streamline RNAi screens by focusing on genes with translational potential to human health and reducing screening efforts by 60%.

In *C. elegans*, homozygous *phb-*1 and *phb-2* deletion mutants are sterile and need to be maintained as balanced heterozygous. Here we show that homozygous *phb-2(tm2998)* mutants have delayed development, a shorter lifespan and a strong induction of the mitochondrial stress response. Studies looking for the molecular mechanism of this response have identified a number of proteins as essential players for the induction of the UPR^mt^. An uncharacterised temperature-sensitive mutant isolated from ethyl methanesulphonate (EMS), *zc32*, that induces the UPR^mt^ under non-permissive temperature (25 °C), was used to screen chromosome I [44]. RNAi depletion of two genes, *lin-35* and *ubl-5*, suppressed induction of P*hsp-6*::GFP and P*hsp-60*::GFP. With the same mutant, three other components of the UPR^mt^ were described, the transcription factors DVE-1 and ATFS-1 and the mitochondrial transporter HAF-1 [45, 49]. HAF-1 was first suggested as an essential upstream component of the UPR^mt^ [49, 50]; however, conditions that induce the UPR^mt^, such as inhibition of mitochondrial protein import [67] or RNAi against the cytochrome *c* oxidase subunit *cco-1*, do not require HAF-1 [50, 52]. Here we demonstrate that, rather than blocking, loss of HAF-1 further induces *hsp-6* expression upon depletion of *phb-1*, *phb-2* or *spg-7* (Fig. 1d and Additional file 1: Figure S1). This data suggests that more studies are required for the complete understanding of the regulation of the mitochondrial stress response. More recently, Shore et al. analysed the induction of different cytoprotective responses such as ER stress, mitochondrial stress, oxidative and osmotic stress, amongst others, upon depletion of 160 genes reported to increase lifespan in *C. elegans* [61]. They identified 42 RNAi clones required for the activation of the UPR^mt^ triggered by antimycin, a chemical that disrupts complex III of the mitochondrial electron transport chain (ETC). In a genome-wide screen, Runkel et al. found 55 genes necessary for the activation of the UPR^mt^ induced by paraquat treatment [60]. Applying our protocol, we describe 208 genes that, when depleted, reduce the mitochondrial stress response in *phb-2* mutants. On comparison with the previously published data, we found 45.5% of genes previously identified to be required for the activation of the UPR^mt^. This big number of additional candidates can be explained by the quantitative nature of the protocol. In addition, genes affecting development might result in reduced UPR^mt^ reporter expression because *hsp-6* expression increases as worms develop. Similarly, it is worth stressing the different experimental conditions; e.g. chemical treatments or a temperature-sensitive mutant versus a mutant of the mitochondrial PHB complex might trigger the mitochondrial stress response through an alternative pathway. In addition to earlier published clones, we identified numerous genes encoding proteins of the two ribosomal subunits, proteins associated with protein transport including nuclear importins, proteasomal subunits and genes involved in mRNA processing, all processes already known to regulate the UPR^mt^. Interestingly, many of the processes mentioned above are related with proteostasis. Certainly, inhibition of translation will reduce the expression of any GFP reporter without having a direct relation with the mitochondrial stress response, and one should investigate in more detail how silencing ribosomal subunits affects the UPR^mt^. Nevertheless, it has been previously described how attenuating cytosolic protein synthesis strongly suppresses age-related mitochondrial degeneration in yeast models, including the pro-ageing prohibitin mutants [68]. In agreement, other studies in flies and worms support the hypothesis that inhibition of cytosolic translation is protective during mitochondrial dysfunction [69, 70].

In addition to genes already described to be regulators of the UPR^mt^, in this study we found new PHB interactors regulating the mitochondrial stress response such us histones *his-67* and *his-68* and the histones modifiers *pcaf-1* (histone acetyltransferase) and *met-1* (histone methyltransferase). Given the already-described role of epigenetic modifications in regulating the UPR^mt^ [71, 72], studying in more detail the involvement of these epigenetic markers would shed light on the molecular mechanism of the mitochondrial stress response and its impact on ageing.

Interestingly, we encountered 303 PHB interactors whose depletion causes a developmental defect. The fact that chromosome I is particularly enriched in essential genes partially explains this high number of interactions [73]. We uncovered 31% of the genes previously described to affect development [56, 62]. As mentioned before, experimental procedures were different and could explain the non-complete replicability: *phb-2* mutants have per se a developmental delay, and RNAi treatment is performed from L2s instead of from eggs. Therefore, L1-acting genes could have been missed. Interestingly, we found several genes involved in fatty acid metabolism whose depletion arrests development of wild-type animals (*ech-1.2*), while others seem to be PHB-specific (*lbp-5, acox-1.3* or *F10G8.9*). Similarly, genes encoding for nuclear pore complex proteins (*npp-2, npp-6* and *npp-7*) and meiotic spindle organisation (*aspm-1*) also delay wild-type development, while genes implicated in DNA replication/repair (*cdt-1, crn-1, msh-6* and *rpa-4*) and chromosome segregation (*icp-1*) seem to be PHB-specific, as no developmental phenotypes have been described in other RNAi screens [74]. It would be highly relevant to study in more detail why genes involved in these processes affect the development of prohibitin mutants.

## Conclusions

The function of many essential genes is not well understood. The method described here combines automated worm sorting and high-content image analysis that can be adapted and applied to any balanced strain carrying a fluorescently labelled balancer and potentially an additional gene expression reporter. Therefore, this method can be instrumental to increase our knowledge on the biology of essential *C. elegans* genes and the generation of genetic interaction networks in which they are involved. Approximately 60% of essential *C. elegans* genes have human orthologues, making the worm relevant to study the function of genes with an impact on human health.

## Methods

A detailed description of the protocol, day by day, can be found in Additional file 3.

### *C. elegans* strains and maintenance

The *C. elegans* strains used in this study were: N2 (wild type); MRS106: *phb-2*(*tm2998*)/*mIn1*[*dpy-10*(*e128*)*mIs*14(P*myo-2*::GFP)]II;*zcIs13*[P*hsp-6::*GFP]V; MRS104: *phb-2*(*tm2998*)/*mIn1*[*dpy-10*(*e128*)*mIs*14(P*myo-2*::GFP)]II;z*cIs13*[P*hsp-60::*GFP]V; MRS50: *phb-2)*(*tm2998*/*mIn1*[*dpy-10*(*e128*)*mIs*14(P*myo-2*::GFP)]II;*haf-1*(*ok705*)IV;*zcIs13*[P*hsp-6::*GFP]V; BR6118: *haf-1*(*ok705*)IV;*zcIs13*[P*hsp-6::*GFP]V; BR6115: *phb-1*(*tm2571*)*I/hT2*[*bli-4(e937)qIs48*(P*myo-2*::GFP)*]*(*I;III*), 10 times outcrossed before introducing the *hT2* balancer; BR6108: *phb-2*(*tm2998*)*/mln1*[*dpy-10*(*e128*) * mIs*14(P*myo-2*::GFP)]*II*, 10 times outcrossed before introducing the *mIn1* balancer; PE255: *feIs5*[P*sur-5::luc+::*GFP*;rol-6*(*su1006*)]*X*; MRS229: *phb-2*(*tm2998*)*/mln1*[*dpy-10*(*e128*)*mIs*14(P*myo-2*::GFP)]*II;feIs5*[P*sur-5::luc+::GFP;rol-6(su1006)*]*X*.

Unless otherwise stated, we cultured the worms according to standard methods [75]. We maintained nematodes at 20 °C on nematode growth media (NGM) agar plates seeded with live *Escherichia coli* OP50 (obtained from the CGC). To obtain synchronised L1 larvae, we collected eggs by hypochlorite treatment and allowed them to hatch and arrest by overnight incubation in M9 at 20 °C with agitation.

### Generation of the OrthoList RNAi sublibrary

Plates from Ahringer’s RNAi library containing the clones of interest (purchased from the Medical Research Council (MRC) GeneService) were replicated to Luria-Bertani  (LB) agar supplemented with ampicillin (100 μg/ml, Sigma-Aldrich) and tetracycline (15 μg/ml, Sigma-Aldrich) using a pin replicator (Boekel) and grown overnight at 37 °C. The day after, the selected clones were inoculated in 1.3 ml of LB supplemented with ampicillin (100 μg/ml, Sigma-Aldrich), tetracycline (15 μg/ml, Sigma-Aldrich) and 8% glycerol in deep well plates (VWR) and incubated overnight at 37 °C with shaking (180 rpm, New Brunswick™ Innova® 44/44R incubator shaker). The last column of the plates was left free for convenient control. The next day, 120 μl of the culture was transferred to microtitre plates using a Precision XS Microplate Sample Processor (Biotek, Winooski, VT, USA) and frozen at −80 °C.

### Preparation of the bacteria

OrthoList RNAi plates were replicated in LB agar, and bacteria were grown overnight at 37 °C. The advantage of growing the bacteria in solid media is the ease of visualising the clones where the bacteria did not grow. If needed, the samples can be kept at 4 °C for 2 days maximum. Next day, we inoculated the RNAi library in 2.2-ml 96-well plates (VWR). Using the pin replicator, we inoculated the bacteria from the LB agar into 1.2 ml of LB supplemented with ampicillin (100 μg/ml, Sigma-Aldrich) and tetracycline (15 μg/ml, Sigma-Aldrich). Positive and negative controls were added in the last column of the plate. We cultured the bacteria overnight at 37 °C with shaking (180 rpm, New Brunswick™ Innova® 44/44R). In order to have fresh cultures, on the day of sorting, we inoculated 100 μl of the O/N cultures in 900 μl of LB supplemented with ampicillin and tetracycline in deep well plates (VWR) and incubated for 3 h at 37 °C with shaking. We added isopropylthio-β-galactoside (IPTG, 1 mM, Sigma-Aldrich) to the wells to induce the expression of the plasmid for 2 h at 37 °C with shaking. Then, we harvested the cultures by centrifugation (10 min, 3200 g, 4 °C, Eppendorf 5810R) and re-suspended the pellets in 250 μl of S-medium supplemented with carbenicillin (25 μg/ml, Sigma-Aldrich), IPTG (1 mM, Sigma-Aldrich) and cholesterol (5 μg/ml, Sigma-Aldrich).

### Worm preparation and sorting

For each round of sorting, we synchronised the worms, obtaining eggs from gravid hermaphrodites by hypochlorite treatment. Briefly, 20 ml of liquid culture with worms suspended in S-medium supplemented with OP50 (30 g/L wet weight) was washed with M9 until the supernatant appeared clear of bacteria. We added bleaching solution and energetically agitated the tubes for 2 min. After centrifugation and removal of the supernatant, we washed the worms with M9. We added a second round of bleaching solution for less than 1 min. We washed the pellets three more times with M9 and filtered them with 40 μm Nylon Cell Strainers (VWR) to remove the possible remains of adult worms. We allowed the embryos to hatch overnight in M9 at 20 °C with shaking (120 rpm, New Brunswick™ Innova® 44/44R). The following day, we placed the starved L1s in S-medium with OP50 (30 g/L) for 48 h at 20 °C with shaking (120 rpm, New Brunswick™ Innova® 44/44R). At this point, the population is heterogeneous, since the homozygous *phb-2(tm2998)* mutants show a developmental delay and are at the second larval stage. Worms were washed out from the OP50 culture by successive centrifugations until the supernatant was clear. We re-suspended worms in M9 supplemented with 0.01% Triton X-100 (T8787, Sigma-Aldrich) to avoid their sticking to the plastic. Next, we sorted 40 worms per well using enhanced mode, with a sort delay (time from analysis of the object to the sort command) of 7 ms and a sort width (drop volume) of 6 ms.

Subsequently, we added to the worms 25 μl of S-medium supplemented with carbenicillin (25 μg/ml, Sigma-Aldrich), IPTG (1 mM, Sigma-Aldrich) and cholesterol (5 μg/ml, Sigma-Aldrich), and then 75 μl of the bacterial culture was added. We incubated the worms for 48 h at 20 °C with shaking (120 rpm, New Brunswick™ Innova® 44/44R) until they reached the young adult stage.

During the sorting, we aimed to keep the sheath flow rate constant at 9.5 ml/min and a worm concentration of 15–20 events per second. At the start of each experiment, a small sample (one single worm in 96 wells) was sorted and visually verified to confirm a correct sorting, that is, correct number of animals and correct selection of the population.

The worm sorter is a pressurised machine, and one should pay attention to any clog that might interfere with the liquid flow from the sample cup to the flow cell.

Every day, before starting, the tubes were cleaned by passing consecutively from the sample cup 10% bleach, water and 70% ethanol, and then rinsing with abundant water. Moreover, all the solutions were passed through a 40 μm Nylon Cell Strainer (VWR) filter.

For imaging in the red channel, we sorted 40 homozygous *phb-2(tm2998)* mutants at the L1 larval stage after overnight starvation, using the COPAS without utilising the Profiler feature, into 96-well plates. Also, approximately 40 synchronised wild-type animals at the L1 stage were pipetted manually into microtitre plates. All worms were grown in 100 μl HT115 (DE3) bacteria containing the empty vector pL4440 supplemented with 100 nM Nile Red and incubated at 20 °C with shaking. The young adults were then imaged.

### Imaging of multiwell plates

In order to have clear images, we washed the plates by sequential flushes of water, shaking to disaggregate the bacteria, sedimentation of the worms and aspiration of the supernatant (EL406 washer dispenser, Biotek). Prior to this, we added 10 μl of tetramisole hydrochloride (100 mM, Sigma-Aldrich) to each well to paralyse the worms. Each well was filled to the brim and sealed with transparent SealPlate (Sigma-Aldrich) to ensure the horizontal meniscus required to give uniform brightfield illumination across each well. We acquired pictures in brightfield, green and/or red channel with the IN Cell Analyzer 2000 (GE Healthcare) using a 2× objective in order to have the entire well in one image. A whole 96-well plate can be imaged in two channels in less than 10 min and in three channels in less than 15 min.

### Data analysis

Our pipeline consists of several filtering steps, quality control and statistical test. First, based on the green head ID, worms with a green pharynx were discarded, as well as worms with a length smaller than 550 μm. In order to remove outliers, the 5th and the 95th percentiles of the distribution were excluded. After filtering, wells with less than five worms were removed from the analysis. Bacteria containing an empty vector, pL4440, were used as negative controls, and *atfs-1(RNAi)*, which suppresses almost completely the UPR^mt^ in *phb-2* mutants, was used as a positive control (Fig. 5a). A quality assay was performed in the control wells: only control wells with mean GFP intensity between 200 and 500 arbitrary units (a.u.) and a coefficient of variation < 0.5 were accepted. If less than two control wells remained accepted, the plate was discarded and the process repeated. In order to make data from different plates comparable, the data was normalised by dividing the GFP value of each worm by the mean of the GFP of the four negative control wells. Finally, statistics were assessed by running an analysis of variance (ANOVA) test followed by a Dunnett’s test. Candidates were defined based on the adjusted *P* value and the fold change (FC) (*P* value < 0.001 and FC < 0.66 or FC > 1.5).

Interaction networks were built using STRING [76], based on predicted and described interactions in different organisms, and functional annotation clustering was performed with DAVID Bioinformatics Resource 6.8 [77].

### Western blot analysis and antibodies

Protein levels were quantified by immunoblot assay. A synchronised population of worms was grown at 20 °C until they reached young adult stage. We transferred the worms to NGM plates without food and allowed them to crawl for half an hour in order to remove excess bacteria; we then collected 40 animals in 15 μl of M9. We added the same volume of 2× sample buffer (100 mM Tris pH 6.8, 0.02% Serva Blue G, 8% sodium dodecyl sulphate (SDS), 24% glycerol and 4% mercapto-ethanol), boiled the sample for 5 min, spun it for 1 min (4 °C) and kept it at −80 °C for 3–5 days. We ran 16 μl of sample in 12% SDS-polyacrylamide gel electrophoresis (PAGE). Following electrophoresis, proteins were transferred to a polyvinylidene fluoride (PVDF) membrane (Immobilon-P, Millipore). We visualised the immunoblots by chemiluminescent detection (Pierce ECL Western Blotting Substrate). We incubated the western blots with Prohibitin_APP-2 antibody (Marta Artal-Sanz’s lab, University Pablo de Olavide, Seville, Spain, Cat. # MRS, RRID: AB_2721134), 1:3000, overnight at 4 °C, and anti-actin (ICN, clone C4) as described [51].

### Luciferase assay to determine developmental rates

We used the reporter strains PE255 and MRS229 to measure larval developmental timing. In order to ensure that all animals started development at the same time, arrested L1s were first manually pipetted to a white 96-well plate, one worm per well, containing 100 μl of S-basal with 100 μM D-luciferin. Development was resumed by addition of 100 μl of S-basal with 20 g/L *E. coli* OP50 and 100 μM D-luciferin. Plates were sealed with a gas-permeable cover (Breathe Easier, Diversified Biotech, Dedham, MA, USA). We measured luminescence in a Berthold Centro LB960 XS3 (Berthold Technologies, Bad Wildbad, Germany) for 1 s, at 5 min intervals. Experiments were done inside temperature-controlled incubators (Panasonic MIR-154). We analysed the raw data from the luminometer as described in Olmedo et al. [48]. Briefly, the raw data was trend-corrected and thresholded using 75% of the moving average to produce a binarised output in order to determine onset and offset of the molts. The data was evaluated for onset and offset of molting by detecting the transitions in the binarised data. To assess statistics, we performed unpaired *t* tests using GraphPad Prism software.

### Lifespan analysis

All lifespans were done at 20 °C. Synchronised eggs were obtained by hypochlorite treatment of adult hermaphrodites and placed on NGM plates containing OP50 *E. coli* bacteria. During the course of the lifespan, we transferred wild-type adult nematodes every day during their reproductive period and afterwards on alternate days. We separated homozygous *phb-1(tm2571)* and *phb-2(tm2998)* mutants from their respective heterozygous populations at L3 stage to a separate plate and transferred them every alternate day. Worms were scored as dead when they stopped responding to prodding, while exploded animals, those exhibiting bagging, protruding gonad or drying out on the edge of the plates were censored. We used GraphPad Prism software to plot survival curves, and we determined significant differences in lifespan by using the log-rank (Mantel-Cox) test.

### Slide imaging

We transferred a semi-synchronous embryo population to NGM plates seeded with the appropriate RNAi bacterial clone. We grew animals at 20 °C until young adult stage, when 30–40 worms were mounted on 2% agarose pads in M9 medium containing 10 mM tetramisole hydrochloride (Sigma) and imaged using an AxioCamMRm camera on a Zeiss ApoTome microscope. In order to remove excess bacteria, we placed worms into NGM plates without food and allowed them to crawl for half an hour. Emission intensity was measured on greyscale images with a pixel depth of 16 bits. Image analysis was performed using the ImageJ software, and we analysed data by one-way ANOVA using GraphPad Prism.

## Additional files

Additional file 1: **Figure S1.** Effect of *haf-1* deletion in the UPR^mt^. **a.** *haf-1(ok705)* deletion further induces P*hsp-6*::GFP expression upon depletion of the PHB complex *(phb-1(RNAi)* or *phb-2(RNAi)*). Bar graphs show quantification of P*hsp-6*::GFP. Day 1 adults are shown. **b.** *haf-1(ok705)* deletion also enhances the expression of the UPR^mt^ reporter P*hsp-6::*GFP in *phb-2(tm2998)* deletion mutants. Day 5 adults were imaged. **c.** *haf-1(ok705)* deletion further induces P*hsp-6::*GFP expression upon RNAi depletion of the mitochondrial AAA protease *(spg-7(RNAi))*. Day 1 adults are shown. All bar graphs show quantification of P*hsp-6*::GFP (mean ± SD); *P* value shown in each panel; two-tailed unpaired *t* test; *n* = 20; two biological repeats, one representative experiment is shown. (PDF 3830 kb)

Additional file 2: **Table S1.** Day-by-day description of the experimental procedure. Schematic view of the 6-day protocol, from worm and bacteria preparation until imaging. (PDF 162 kb)

Additional file 3: Detailed description of the protocol. Report of the strain and reagents used, in addition to a more exhaustive explanation in the methodology followed in the RNAi study. (DOCX 21 kb)

Additional file 4: Breakdown of the image analysis protocol for green images. (XLSX 33 kb)

Additional file 5: Raw data of P*hsp-6*::GFP reporter measurements. Gene-by-gene data of the fold change (FC) and adjusted *P* value (adj.pvalue) obtained after data analysis. RNAi clones appear ordered by their position in the OrthoList RNAi sublibrary, with the GenePair name as well as the common gene name. In sheet 2 is the list of genes already described to regulate the UPR^mt^ in previous screens. (XLSX 102 kb)

Additional file 6: Raw data of size measurements. Gene-by-gene data of the fold change (FC) and adjusted *P* value (adj.pvalue) obtained after data analysis. RNAi clones appear ordered by their position in the OrthoList RNAi sublibrary, with the GenePair name as well as the common gene name. (XLSX 112 kb)

Additional file 7: **Figure S2.** Outline of the combined green/red image analysis for balanced mutants. Briefly, post well segmentation in the green channel, the worms are segmented after enhancing the contrast in the Brightfield channel (*image 1*). Acceptance criteria (Additional File 8) are applied to remove artefacts (blue accepted, yellow rejected). The worm mask is transferred to the *green image* to identify, if needed, worms with green heads based on the green head ID (*image 2* and *image 3*). With the final worm mask dilated (*image 5*), the dilated region around the worm is used to calculate the immediate background intensity. Finally, the targets are linked together to get all three measurement regions (*Dilated worms*, *Worm minus Green head* and *Final worm mask*) together in one target (Fig. 4). As mentioned earlier, the software links targets, two at a time, and there must be at least one pixel overlap to achieve a linkage. (PDF 7627 kb)

Additional file 8: Breakdown of image analysis protocol for green and red images. (XLSX 34 kb)

Additional file 9: Breakdown of CellProfiler protocol for green and red images. (PDF 93 kb)

## References

[1] Dickerson JE, Zhu A, Robertson DL, Hentges KE (2011). Defining the role of essential genes in human disease. PLoS One.

[2] Costanzo M, VanderSluis B, Koch EN, Baryshnikova A, Pons C, Tan G, Wang W, Usaj M, Hanchard J, Lee SD (2016). A global genetic interaction network maps a wiring diagram of cellular function. Science.

[3] Kemphues K. Essential genes. WormBook. 2005:1–7. 10.1895/wormbook.1.57.1 PMC4781014 18023123

[4] Ramani AK, Chuluunbaatar T, Verster AJ, Na H, Vu V, Pelte N, Wannissorn N, Jiao A, Fraser AG (2012). The majority of animal genes are required for wild-type fitness. Cell.

[5] Housden BE, Muhar M, Gemberling M, Gersbach CA, Stainier DY, Seydoux G, Mohr SE, Zuber J, Perrimon N (2017). Loss-of-function genetic tools for animal models: cross-species and cross-platform differences. Nat Rev Genet.

[6] Mok CA, Au V, Thompson OA, Edgley ML, Gevirtzman L, Yochem J, Lowry J, Memar N, Wallenfang MR, Rasoloson D (2017). MIP-MAP: high throughput mapping of Caenorhabditis elegans temperature-sensitive mutants via molecular inversion probes. Genetics.

[7] O'Rourke SM, Carter C, Carter L, Christensen SN, Jones MP, Nash B, Price MH, Turnbull DW, Garner AR, Hamill DR (2011). A survey of new temperature-sensitive, embryonic-lethal mutations in C. elegans: 24 alleles of thirteen genes. PLoS One.

[8] Jaramillo-Lambert A, Fuchsman AS, Fabritius AS, Smith HE, Golden A (2015). Rapid and efficient identification of Caenorhabditis elegans legacy mutations using Hawaiian SNP-based mapping and whole-genome sequencing. G3 (Bethesda).

[9] Lowry J, Yochem J, Chuang CH, Sugioka K, Connolly AA, Bowerman B (2015). High-throughput cloning of temperature-sensitive Caenorhabditis elegans mutants with adult syncytial germline membrane architecture defects. G3 (Bethesda).

[10] Chen X, Li M, Feng X, Guang S (2015). Targeted chromosomal translocations and essential gene knockout using CRISPR/Cas9 technology in Caenorhabditis elegans. Genetics.

[11] Edgley ML, Baillie DL, Riddle DL, Rose AM. Genetic balancers. WormBook. 2006:1–32. 10.1895/wormbook.1.89.1 PMC4781404 18050450

[12] Iwata S, Yoshina S, Suehiro Y, Hori S, Mitani S (2016). Engineering new balancer chromosomes in C. elegans via CRISPR/Cas9. Sci Rep.

[13] Pulak R (2006). Techniques for analysis, sorting, and dispensing of C. elegans on the COPAS flow-sorting system. Methods Mol Biol.

[14] Latorre I, Chesney MA, Garrigues JM, Stempor P, Appert A, Francesconi M, Strome S, Ahringer J (2015). The DREAM complex promotes gene body H2A.Z for target repression. Genes Dev.

[15] Ruegger S, Miki TS, Hess D, Grosshans H (2015). The ribonucleotidyl transferase USIP-1 acts with SART3 to promote U6 snRNA recycling. Nucleic Acids Res.

[16] Doitsidou M, Flames N, Lee AC, Boyanov A, Hobert O (2008). Automated screening for mutants affecting dopaminergic-neuron specification in C. elegans. Nat Methods.

[17] O'Reilly LP, Long OS, Cobanoglu MC, Benson JA, Luke CJ, Miedel MT, Hale P, Perlmutter DH, Bahar I, Silverman GA (2014). A genome-wide RNAi screen identifies potential drug targets in a C. elegans model of alpha1-antitrypsin deficiency. Hum Mol Genet.

[18] Squiban B, Belougne J, Ewbank J, Zugasti O (2012). Quantitative and automated high-throughput genome-wide RNAi screens in C. elegans. J Vis Exp.

[19] Benson JA, Cummings EE, O'Reilly LP, Lee MH, Pak SC (2014). A high-content assay for identifying small molecules that reprogram C. elegans germ cell fate. Methods.

[20] Gosai SJ, Kwak JH, Luke CJ, Long OS, King DE, Kovatch KJ, Johnston PA, Shun TY, Lazo JS, Perlmutter DH (2010). Automated high-content live animal drug screening using C. elegans expressing the aggregation prone serpin alpha1-antitrypsin Z. PLoS One.

[21] Moy TI, Conery AL, Larkins-Ford J, Wu G, Mazitschek R, Casadei G, Lewis K, Carpenter AE, Ausubel FM (2009). High-throughput screen for novel antimicrobials using a whole animal infection model. ACS Chem Biol.

[22] O'Rourke EJ, Conery AL, Moy TI (2009). Whole-animal high-throughput screens: the C. elegans model. Methods Mol Biol.

[23] Wahlby C, Kamentsky L, Liu ZH, Riklin-Raviv T, Conery AL, O'Rourke EJ, Sokolnicki KL, Visvikis O, Ljosa V, Irazoqui JE (2012). An image analysis toolbox for high-throughput C. elegans assays. Nat Methods.

[24] Artal-Sanz M, de Jong L, Tavernarakis N (2006). Caenorhabditis elegans: a versatile platform for drug discovery. Biotechnol J.

[25] Burns AR, Kwok TC, Howard A, Houston E, Johanson K, Chan A, Cutler SR, McCourt P, Roy PJ (2006). High-throughput screening of small molecules for bioactivity and target identification in Caenorhabditis elegans. Nat Protoc.

[26] Giacomotto J, Segalat L, Carre-Pierrat M, Gieseler K (2012). Caenorhabditis elegans as a chemical screening tool for the study of neuromuscular disorders. Manual and semi-automated methods. Methods.

[27] Lehner B, Tischler J, Fraser AG (2006). RNAi screens in Caenorhabditis elegans in a 96-well liquid format and their application to the systematic identification of genetic interactions. Nat Protoc.

[28] Maglioni S, Arsalan N, Ventura N (2016). C. elegans screening strategies to identify pro-longevity interventions. Mech Ageing Dev.

[29] O'Reilly LP, Knoerdel RR, Silverman GA, Pak SC (2016). High-throughput, liquid-based genome-wide RNAi screening in C. elegans. Methods Mol Biol.

[30] Carpenter AE, Jones TR, Lamprecht MR, Clarke C, Kang IH, Friman O, Guertin DA, Chang JH, Lindquist RA, Moffat J (2006). CellProfiler: image analysis software for identifying and quantifying cell phenotypes. Genome Biol.

[31] Wahlby C, Conery AL, Bray MA, Kamentsky L, Larkins-Ford J, Sokolnicki KL, Veneskey M, Michaels K, Carpenter AE, O'Rourke EJ (2014). High- and low-throughput scoring of fat mass and body fat distribution in C. elegans. Methods.

[32] Back JW, Sanz MA, De Jong L, De Koning LJ, Nijtmans LG, De Koster CG, Grivell LA, Van Der Spek H, Muijsers AO (2002). A structure for the yeast prohibitin complex: structure prediction and evidence from chemical crosslinking and mass spectrometry. Protein Sci.

[33] Artal-Sanz M, Tsang WY, Willems EM, Grivell LA, Lemire BD, van der Spek H, Nijtmans LG (2003). The mitochondrial prohibitin complex is essential for embryonic viability and germline function in Caenorhabditis elegans. J Biol Chem.

[34] Berger KH, Yaffe MP (1998). Prohibitin family members interact genetically with mitochondrial inheritance components in Saccharomyces cerevisiae. Mol Cell Biol.

[35] Artal-Sanz M, Tavernarakis N (2009). Prohibitin and mitochondrial biology. Trends Endocrinol Metab.

[36] Merkwirth C, Langer T (2009). Prohibitin function within mitochondria: essential roles for cell proliferation and cristae morphogenesis. Biochim Biophys Acta.

[37] Osman C, Haag M, Potting C, Rodenfels J, Dip PV, Wieland FT, Brugger B, Westermann B, Langer T (2009). The genetic interactome of prohibitins: coordinated control of cardiolipin and phosphatidylethanolamine by conserved regulators in mitochondria. J Cell Biol.

[38] Merkwirth C, Dargazanli S, Tatsuta T, Geimer S, Lower B, Wunderlich FT, von Kleist-Retzow JC, Waisman A, Westermann B, Langer T (2008). Prohibitins control cell proliferation and apoptosis by regulating OPA1-dependent cristae morphogenesis in mitochondria. Genes Dev.

[39] Nijtmans LG, Artal SM, Grivell LA, Coates PJ (2002). The mitochondrial PHB complex: roles in mitochondrial respiratory complex assembly, ageing and degenerative disease. Cell Mol Life Sci.

[40] Thuaud F, Ribeiro N, Nebigil CG, Desaubry L (2013). Prohibitin ligands in cell death and survival: mode of action and therapeutic potential. Chem Biol.

[41] Merkwirth C, Martinelli P, Korwitz A, Morbin M, Bronneke HS, Jordan SD, Rugarli EI, Langer T (2012). Loss of prohibitin membrane scaffolds impairs mitochondrial architecture and leads to tau hyperphosphorylation and neurodegeneration. PLoS Genet.

[42] Nijtmans LG, de Jong L, Artal Sanz M, Coates PJ, Berden JA, Back JW, Muijsers AO, van der Spek H, Grivell LA (2000). Prohibitins act as a membrane-bound chaperone for the stabilization of mitochondrial proteins. EMBO J.

[43] Wei Y, Chiang WC, Sumpter R, Mishra P, Levine B (2017). Prohibitin 2 is an inner mitochondrial membrane mitophagy receptor. Cell.

[44] Benedetti C, Haynes CM, Yang Y, Harding HP, Ron D (2006). Ubiquitin-like protein 5 positively regulates chaperone gene expression in the mitochondrial unfolded protein response. Genetics.

[45] Haynes CM, Petrova K, Benedetti C, Yang Y, Ron D (2007). ClpP mediates activation of a mitochondrial unfolded protein response in C. elegans. Dev Cell.

[46] Yoneda T, Benedetti C, Urano F, Clark SG, Harding HP, Ron D (2004). Compartment-specific perturbation of protein handling activates genes encoding mitochondrial chaperones. J Cell Sci.

[47] Artal-Sanz M, Tavernarakis N (2009). Prohibitin couples diapause signalling to mitochondrial metabolism during ageing in C. elegans. Nature.

[48] Olmedo M, Geibel M, Artal-Sanz M, Merrow M (2015). A high-throughput method for the analysis of larval developmental phenotypes in Caenorhabditis elegans. Genetics.

[49] Haynes CM, Yang Y, Blais SP, Neubert TA, Ron D (2010). The matrix peptide exporter HAF-1 signals a mitochondrial UPR by activating the transcription factor ZC376.7 in C. elegans. Mol Cell.

[50] Nargund AM, Pellegrino MW, Fiorese CJ, Baker BM, Haynes CM (2012). Mitochondrial import efficiency of ATFS-1 regulates mitochondrial UPR activation. Science.

[51] Gatsi R, Schulze B, Rodriguez-Palero MJ, Hernando-Rodriguez B, Baumeister R, Artal-Sanz M (2014). Prohibitin-mediated lifespan and mitochondrial stress implicate SGK-1, insulin/IGF and mTORC2 in C. elegans. PLoS One.

[52] Bennett CF, Vander Wende H, Simko M, Klum S, Barfield S, Choi H, Pineda VV, Kaeberlein M (2014). Activation of the mitochondrial unfolded protein response does not predict longevity in Caenorhabditis elegans. Nat Commun.

[53] Houtkooper RH, Mouchiroud L, Ryu D, Moullan N, Katsyuba E, Knott G, Williams RW, Auwerx J (2013). Mitonuclear protein imbalance as a conserved longevity mechanism. Nature.

[54] Shaye DD, Greenwald I (2011). OrthoList: a compendium of C. elegans genes with human orthologs. PLoS One.

[55] Timmons L, Fire A (1998). Specific interference by ingested dsRNA. Nature.

[56] Kamath RS, Fraser AG, Dong Y, Poulin G, Durbin R, Gotta M, Kanapin A, Le Bot N, Moreno S, Sohrmann M (2003). Systematic functional analysis of the Caenorhabditis elegans genome using RNAi. Nature.

[57] Rual JF, Ceron J, Koreth J, Hao T, Nicot AS, Hirozane-Kishikawa T, Vandenhaute J, Orkin SH, Hill DE, van den Heuvel S (2004). Toward improving Caenorhabditis elegans phenome mapping with an ORFeome-based RNAi library. Genome Res.

[58] Gerstein MB, Lu ZJ, Van Nostrand EL, Cheng C, Arshinoff BI, Liu T, Yip KY, Robilotto R, Rechtsteiner A, Ikegami K (2010). Integrative analysis of the Caenorhabditis elegans genome by the modENCODE project. Science.

[59] Qu W, Ren C, Li Y, Shi J, Zhang J, Wang X, Hang X, Lu Y, Zhao D, Zhang C (2011). Reliability analysis of the Ahringer Caenorhabditis elegans RNAi feeding library: a guide for genome-wide screens. BMC Genomics.

[60] Runkel ED, Liu S, Baumeister R, Schulze E (2013). Surveillance-activated defenses block the ROS-induced mitochondrial unfolded protein response. PLoS Genet.

[61] Shore DE, Carr CE, Ruvkun G (2012). Induction of cytoprotective pathways is central to the extension of lifespan conferred by multiple longevity pathways. PLoS Genet.

[62] Fraser AG, Kamath RS, Zipperlen P, Martinez-Campos M, Sohrmann M, Ahringer J (2000). Functional genomic analysis of C. elegans chromosome I by systematic RNA interference. Nature.

[63] Dupuy D, Bertin N, Hidalgo CA, Venkatesan K, Tu D, Lee D, Rosenberg J, Svrzikapa N, Blanc A, Carnec A (2007). Genome-scale analysis of in vivo spatiotemporal promoter activity in Caenorhabditis elegans. Nat Biotechnol.

[64] Watson E, MacNeil LT, Arda HE, Zhu LJ, Walhout AJM (2013). Integration of metabolic and gene regulatory networks modulates the C. elegans dietary response. Cell.

[65] Maia AF, Tanenbaum ME, Galli M, Lelieveld D, Egan DA, Gassmann R, Sunkel CE, van den Heuvel S, Medema RH (2015). Genome-wide RNAi screen for synthetic lethal interactions with the C. elegans kinesin-5 homolog BMK-1. Sci Data.

[66] Zugasti O, Thakur N, Belougne J, Squiban B, Kurz CL, Soule J, Omi S, Tichit L, Pujol N, Ewbank JJ (2016). A quantitative genome-wide RNAi screen in C. elegans for antifungal innate immunity genes. BMC Biol.

[67] Nargund AM, Fiorese CJ, Pellegrino MW, Deng P, Haynes CM (2015). Mitochondrial and nuclear accumulation of the transcription factor ATFS-1 promotes OXPHOS recovery during the UPR(mt). Mol Cell.

[68] Wang X, Zuo X, Kucejova B, Chen XJ (2008). Reduced cytosolic protein synthesis suppresses mitochondrial degeneration. Nat Cell Biol.

[69] Baker BM, Nargund AM, Sun T, Haynes CM (2012). Protective coupling of mitochondrial function and protein synthesis via the eIF2alpha kinase GCN-2. PLoS Genet.

[70] Liu S, Lu B (2010). Reduction of protein translation and activation of autophagy protect against PINK1 pathogenesis in Drosophila melanogaster. PLoS Genet.

[71] Merkwirth C, Jovaisaite V, Durieux J, Matilainen O, Jordan SD, Quiros PM, Steffen KK, Williams EG, Mouchiroud L, Tronnes SU (2016). Two conserved histone demethylases regulate mitochondrial stress-induced longevity. Cell.

[72] Tian Y, Garcia G, Bian Q, Steffen KK, Joe L, Wolff S, Meyer BJ, Dillin A (2016). Mitochondrial stress induces chromatin reorganization to promote longevity and UPR(mt). Cell.

[73] Chu JS, Chua SY, Wong K, Davison AM, Johnsen R, Baillie DL, Rose AM (2014). High-throughput capturing and characterization of mutations in essential genes of Caenorhabditis elegans. BMC Genomics.

[74] Wormbase. http://www.wormbase.org.

[75] Brenner S (1974). The genetics of Caenorhabditis elegans. Genetics.

[76] Szklarczyk D, Morris JH, Cook H, Kuhn M, Wyder S, Simonovic M, Santos A, Doncheva NT, Roth A, Bork P (2017). The STRING database in 2017: quality-controlled protein-protein association networks, made broadly accessible. Nucleic Acids Res.

[77] Huang da W, Sherman BT, Lempicki RA. Systematic and integrative analysis of large gene lists using DAVID bioinformatics resources. Nat Protoc 2009;4(1):44–57. 10.1038/nprot.2008.211 19131956

