# Supplementary figures and images for: Combined flow cytometry and high-throughput image analysis for the study of essential genes in Caenorhabditis elegans
Source: BMC Biol. 2018 Mar 29;16:36. doi: 10.1186/s12915-018-0496-5 (PMC5875015; doi:10.1186/s12915-018-0496-5)

# Additional file 1: Figure S1

**a.**

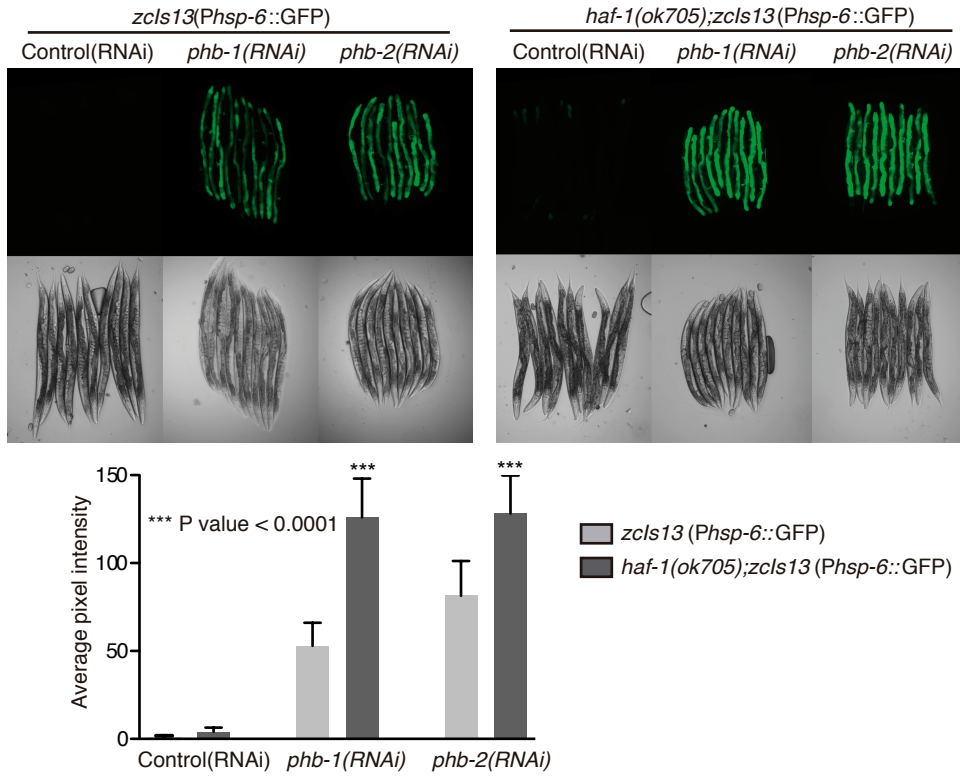

**b.**

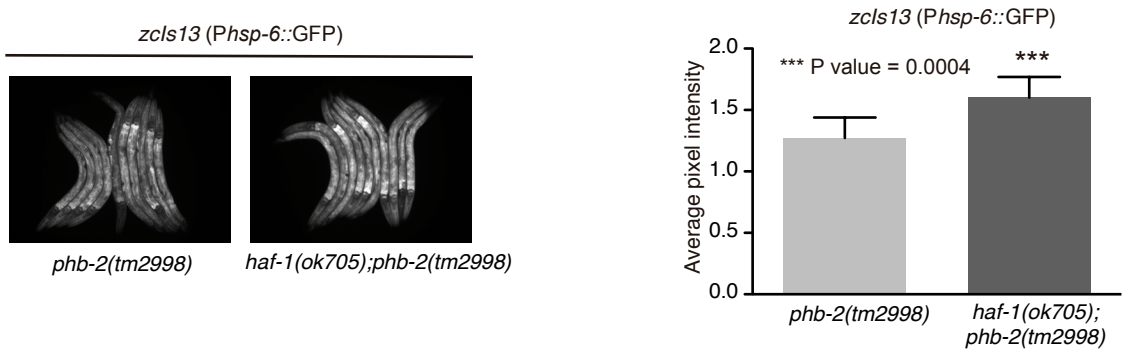

**c.**

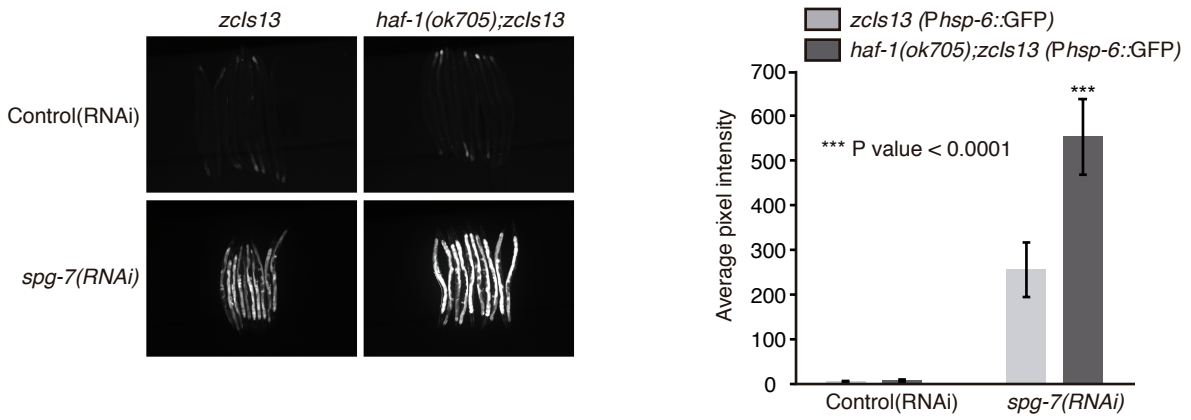

Supplement: Supplementary file 1 — Figure S1. Effect of haf-1 deletion in the UPRmt. a. haf-1(ok705) deletion further induces Phsp-6::GFP expression upon depletion of the PHB complex (phb-1(RNAi) or phb-2(RNAi)). Bar graphs show quantification of Phsp-6::GFP. Day 1 adults are shown. b. haf-1(ok705) deletion also enhances the expression of the UPRmt reporter Phsp-6::GFP in phb-2(tm2998) deletion mutants. Day 5 adults were imaged. c. haf-1(ok705) deletion further induces Phsp-6::GFP expression upon RNAi depletion of the mitochondrial AAA protease (spg-7(RNAi)). Day 1 adults are shown. All bar graphs show quantification of Phsp-6::GFP (mean ± SD); P value shown in each panel; two-tailed unpaired t test; n = 20; two biological repeats, one representative experiment is shown. (PDF 3830 kb) [file 12915_2018_496_MOESM1_ESM.pdf]

## Additional file 7: Figure S2

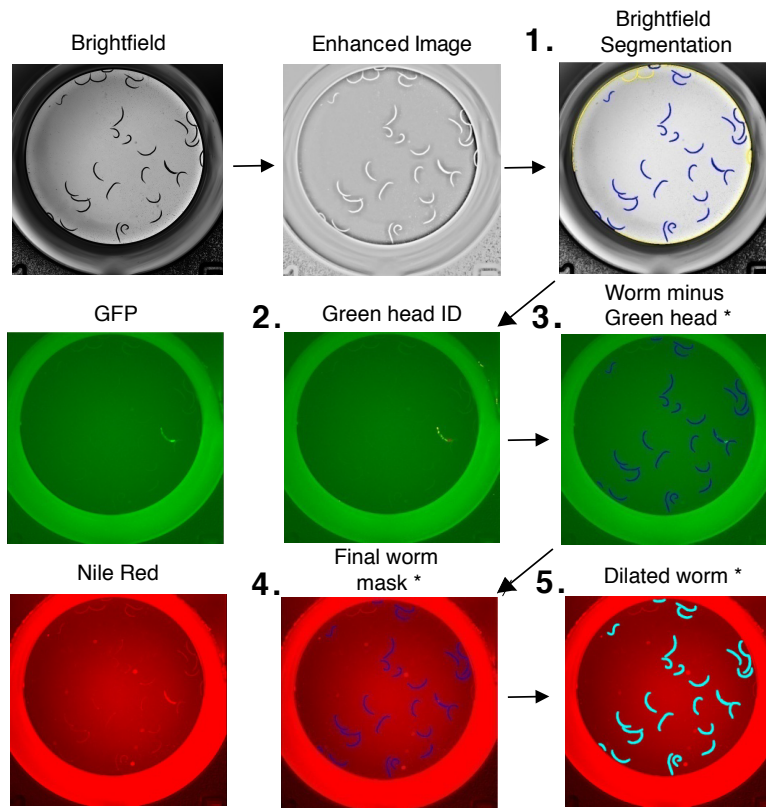

\*Target Linking

Supplement: Supplementary file 7 — Figure S2. Outline of the combined green/red image analysis for balanced mutants. Briefly, post well segmentation in the green channel, the worms are segmented after enhancing the contrast in the Brightfield channel (image 1). Acceptance criteria (Additional File 8) are applied to remove artefacts (blue accepted, yellow rejected). The worm mask is transferred to the green image to identify, if needed, worms with green heads based on the green head ID (image 2 and image 3). With the final worm mask dilated (image 5), the dilated region around the worm is used to calculate the immediate background intensity. Finally, the targets are linked together to get all three measurement regions (Dilated worms, Worm minus Green head and Final worm mask) together in one target (Fig. 4). As mentioned earlier, the software links targets, two at a time, and there must be at least one pixel overlap to achieve a linkage. (PDF 7627 kb) [file 12915_2018_496_MOESM7_ESM.pdf]
