# Supplementary material for: Combined flow cytometry and high-throughput image analysis for the study of essential genes in Caenorhabditis elegans
Source: BMC Biol. 2018 Mar 29;16:36. doi: 10.1186/s12915-018-0496-5 (PMC5875015; doi:10.1186/s12915-018-0496-5)
Supplement: Supplementary file 2 — Table S1. Day-by-day description of the experimental procedure. Schematic view of the 6-day protocol, from worm and bacteria preparation until imaging. (PDF 162 kb) [file 12915_2018_496_MOESM2_ESM.pdf]

Additional file 2: Table S1

	Preparation of the worms	Preparation of the bacteria
Day 1	Bleaching *phb-2(tm2998)/mIn1*;P*hsp-6*::GFP	Inoculate RNAi library in LB agar<br>O/N 37ºC
Day 2	Place starved L1s in liquid OP50<br>20ºC, 120 rpm	
Day 3		Replicate RNAi library in deep well plates<br>O/N 37ºC, 180 rpm
Day 4	Sorting L2s *phb-2(tm2998);*P*hsp-6*::GFP	Bacterial culture<br>and resuspend in complete S medium
Day 5	Incubate 20ºC, 120 rpm	
Day 6	Imaging	
