# Supplementary material for: Combined flow cytometry and high-throughput image analysis for the study of essential genes in Caenorhabditis elegans
Source: BMC Biol. 2018 Mar 29;16:36. doi: 10.1186/s12915-018-0496-5 (PMC5875015; doi:10.1186/s12915-018-0496-5)
Supplement: Supplementary file 3 — Detailed description of the protocol. Report of the strain and reagents used, in addition to a more exhaustive explanation in the methodology followed in the RNAi study. (DOCX 21 kb) [file 12915_2018_496_MOESM3_ESM.docx]

DETAILED EXPERIMENTAL WORKFLOW

Screening strain and reagents:

- *phb-2(tm2998)/mIn1[dpy-10(e128)mIs14(*P*myo-2*::GFP*)]II;*P*hsp6*::GFP mutants
- RNAi library based on the compendium of the *C. elegans* genes sharing homologues in humans[1].
- Last column of the plates reserved for controls: Empty feeding vector, pL4440, as negative control and *atfs-1(RNAi)*, that suppresses almost completely the UPR^mt^, as positive.
- LB (Luria-Bertani) media, solid and liquid, supplemented with ampicillin 100μg/ml and tetracycline 15 μg/ml, both from Sigma-Aldrich.
- M9 buffer:22 mM KH_2_PO_4_, 33.7 mMNa_2_HPO_4_, 85.6 mMNaCl, 1 mMMgSO_4_.
- S medium buffer (potassium phosphate pH6 50mM, potassium citrate pH6 10mM, NaCl 100mM, CaCl_2_ 3mM, MgSO_4_ 3mM, trace metal solution) supplemented with carbenicillin 25 μg/ml, IPTG 1 mM and cholesterol 5 μg/ml, all of them from Sigma-Aldrich.
- Bleaching solution (2.75 ml H_2_O, 2 ml commercial bleach, 0.250 ml 5 M KOH)
- 1mM IPTG from Sigma-Aldrich to induce the expression of the dsRNA in the bacteria
- Triton X-100 (T8787) from Sigma-Aldrich
- Tetramisole hydrochloride (L9756) from Sigma-Aldrich

Day 1:

- RNAi library replication: The RNAi library was frozen in glycerol supplemented LB in microtiter plates. The last column of the plates was left free to add the pertinent controls. Using a pin replicator (BOEKEL), we replicated the plate in LB agar supplemented with ampicillin (100μg/ml - Sigma-Aldrich) and tetracycline (15 μg/ml – Sigma-Aldrich). The advantage of growing the bacteria in solid media is the ease to visualize the wells where the bacteria did not grow. Bacteria was grown overnight at 37ºC and then kept at 4ºC.
- Synchronization of *phb-2(tm2998)/mIn1;*P*hsp-6*::GFPmutants: The worms were synchronized as previously described [2]. Briefly, 20 ml of liquid culture with worms in OP50 (30 g/l) were washed with M9 until the supernatant appeared clear of bacteria. Bleaching solution was added and tubes were energetically agitated for 2 minutes. After centrifugation and removal of the supernatant, the worms were washed with M9. A second round of bleaching solution was added for less than 1 minute. The pellet was then washed three more times with M9 and filtered with 40 μm Nylon Cell Strainers (VWR) to remove the possible remains of adult worms. Embryos were allowed to hatch overnight in M9 at 20ºC with shaking (120rpm - New Brunswick™ Innova^®^ 44/44R).

Day 2:

- Starved *phb-2(tm2998)/mIn1;*P*hsp-6*::GFP L1s were placed in liquid OP50 (30 g/l) for 48 hours at 20ºC with shaking, (120rpm - New Brunswick™ Innova^®^ 44/44R), until they reached L2 stage.

Day 3:

- Inoculation of the RNAi library in 2.2ml 96 well plates: Using the pin replicator (BOEKEL) we inoculated the bacteria from LB agar in 1.2 ml of LB supplemented with ampicillin (100μg/ml - Sigma-Aldrich) and tetracycline (15 μg/ml – Sigma-Aldrich) in deep well plates (VWR). Positive and negative controls were added in the last column of the plate. The bacterial cultures were grown overnight at 37ºC with shaking (180rpm - New Brunswick™ Innova^®^ 44/44R).

Day 4:

- Preparation of the bacteria: 100 μl of the O/N cultures were inoculated in 900 μl of LB supplemented with ampicillin (100μg/ml - Sigma-Aldrich) and tetracycline (15 μg/ml – Sigma-Aldrich) in deep well plates (VWR) and incubated for 3 hours at 37ºC with shaking (180rpm - New Brunswick™ Innova^®^ 44/44R). IPTG (1mM– Sigma-Aldrich) was added to the wells in order to induce the expression of the plasmid for 2 hours at 37ºC with shaking (180rpm - New Brunswick™ Innova^®^ 44/44R). The cultures were harvested by centrifugation (3200g during 10 minutes at 4ºC – Eppendorf 5810R) and pellets were resuspended in 250 μl of S-medium supplemented with carbenicillin (25 μg/ml – Sigma-Aldrich), IPTG (1 mM – Sigma-Aldrich) and cholesterol (5 μg/ml – Sigma-Aldrich).
- Preparation of the worms: Worms were washed out from the OP50 culture by successive centrifugations until the supernatant was clear. Worms were resuspended in M9 supplemented with Triton X-100 (Sigma-Aldrich) to a final concentration of 0.01% to avoid adherence to the plastic.
- Sorting of the worms: 40 homozygous L2s were sorted in each well in a volume of 40 μl, 25 μl of S medium supplemented with carbenicillin (25 μg/ml – Sigma-Aldrich), IPTG(1 mM – Sigma-Aldrich) and cholesterol (5 μg/ml – Sigma-Aldrich) were added to the worms. Finally, 75 μl of the bacterial culture was added.
- Worms were incubated for 48 hours at 20ºC with shaking, (120rpm - New Brunswick™ Innova^®^ 44/44R) until they reached young adult stage.

Day 6:

- Imaging of the plates: In order to have clear images, the plates were washed by sequential flush of water, shaken to disaggregate the bacteria, sedimentation of the worms and aspiration of the supernatant (EL406 washer dispenser, BioTek). Prior to this, 10 μl of tetramisolehydrochloride (100 mM - Sigma-Aldrich) was added to each well to paralyze the worms. Pictures in brightfield and green channel were acquired using the IN Cell Analyzer 2000 (GE Healthcare). The 2x objective was used in order to have the entire well in one image.

1. Shaye DD, Greenwald I: **OrthoList: a compendium of C. elegans genes with human orthologs**. *PloS one* 2011, **6**(5):e20085.

2. Stiernagle T: **Maintenance of C. elegans**. *WormBook* 2006:1-11.
