# Supplementary material for: Combined flow cytometry and high-throughput image analysis for the study of essential genes in Caenorhabditis elegans
Source: BMC Biol. 2018 Mar 29;16:36. doi: 10.1186/s12915-018-0496-5 (PMC5875015; doi:10.1186/s12915-018-0496-5)
Supplement: Supplementary file 9 — Breakdown of CellProfiler protocol for green and red images. (PDF 93 kb) [file 12915_2018_496_MOESM9_ESM.pdf]

CellProfiler Pipeline: http://www.cellprofiler.org Version: 3

<u>IMPUT MODULES</u>

<u>Module 1:</u> Images: 'To begin creating your project, use the Images module to compile a list of files and/or folders that you want to analyse. You can also specify a set of rules to include only the desired files in your selected folders.
Filter images? : Images only

<u>Module 2:</u> Metadata: 'The Metadata module optionally allows you to extract information describing your images (i.e, metadata) which will be stored along with your measurements. This information can be contained in the file name and/or location, or in an external file.
Extract metadata? : No
Metadata data type: Text
Metadata types: {}
Extraction method count: 1
Metadata extraction method: Extract from file/folder names
Metadata source: File name
Regular expression:^(?P<**Plate**>.*)_(?P<**Well**>\x5BA-P\x5D\x5B0-9\x5D{2})_s(?P<**Site**>\x5B0-9\x5D)_w(?P<**ChannelNumber**>\x5B0-9\x5D)
Regular expression:(?P<**Date**>\x5B0-9\x5D{4}_\x5B0-9\x5D{2}_\x5B0-9\x5D{2})$
Extract metadata from: All images
Select the filtering criteria: and (file does contain "")
Metadata file location:
Match file and image metadata:\x5B\x5D
Use case insensitive matching? : No

<u>Module 3:</u> Names And Types: 'The Names And Types module allows you to assign a meaningful name to each image by which other modules will refer to it.
Assign a name to: Images matching rules
Select the image type: Grayscale image
Name to assign these images: DNA
Match metadata:\x5B\x5D
Image set matching method: Order
Set intensity range from: Image metadata
Assignments count: 3
Single images count: 0
Maximum intensity: 255.0
Select the rule criteria: and (file does contain "Cy3")
Name to assign these images: Cy3
Name to assign these objects: Cell
Select the image type: Grayscale image
Set intensity range from: Image metadata
Retain outlines of loaded objects? : No
Name the outline image: LoadedOutlines
Maximum intensity: 255.0

Select the rule criteria: and (file does contain "FITC")
Name to assign these images: FITC
Name to assign these objects: Nucleus
Select the image type: Grayscale image
Set intensity range from: Image metadata
Retain outlines of loaded objects? : No
Name the outline image: LoadedOutlines
Maximum intensity: 255.0
Select the rule criteria: and (file does contain "Brightfield")
Name to assign these images: Brightfield
Name to assign these objects: Cytoplasm
Select the image type: Grayscale image
Set intensity range from: Image metadata
Retain outlines of loaded objects? : No
Name the outline image: LoadedOutlines
Maximum intensity: 255.0

<u>Module 4:</u> Groups: 'The Groups module optionally allows you to split your list of images into image subsets (groups) which will be processed independently of each other. Examples of groupings include screening batches, microtiter plates, time-lapse movies, etc.
Do you want to group your images? : No
grouping metadata count: 1
Metadata category: None

<u>ANALYSIS MODULES</u>

<u>Module 5:</u> Image Math:
Operation: Invert
Raise the power of the result by: 1.0
Multiply the result by: 1.0
Add to result: 0.0
Set values less than 0 equal to 0? : Yes
Set values greater than 1 equal to 1? : Yes
Ignore the image masks? : No
Name the output image: InvertedFITC
Image or measurement? : Image
Select the first image: FITC
Multiply the first image by: 1.0
Measurement:
Image or measurement? : Image
Select the second image:
Multiply the second image by: 1.0
Measurement:

<u>Module 6:</u> Identify Primary Objects:
Select the input image: InvertedFITC
Name the primary objects to be identified: Well_FITC_small
Typical diameter of objects, in pixel units (Min, Max): 1500,2000

Discard objects outside the diameter range? : Yes
Try to merge too small objects with nearby larger objects? : No
Discard objects touching the border of the image? : Yes
Method to distinguish clumped objects: None
Method to draw dividing lines between clumped objects: Intensity
Size of smoothing filter: 10
Suppress local maxima that are closer than this minimum allowed distance: 7.0
Speed up by using lower-resolution image to find local maxima? : Yes
Name the outline image: Primary Outlines
Fill holes in identified objects? : After both thresholding and declumping
Automatically calculate size of smoothing filter for declumping? : Yes
Automatically calculate minimum allowed distance between local maxima? : Yes
Retain outlines of the identified objects? : No
Automatically calculate the threshold using the Otsu method? : Yes
Enter Laplacian of Gaussian threshold: 0.5
Automatically calculate the size of objects for the Laplacian of Gaussian filter? : Yes
Enter LoG filter diameter: 5.0
Handling of objects if excessive number of objects identified: Continue
Maximum number of objects: 500
Threshold setting version: 1
Threshold strategy: Global
Thresholding method: Otsu
Select the smoothing method for thresholding: No smoothing
Threshold smoothing scale: 1.0
Threshold correction factor: 1.0
Lower and upper bounds on threshold: 0.0, 1.0
Approximate fraction of image covered by objects? : 0.01
Manual threshold: 0.04
Select the measurement to threshold with: None
Select binary image: None
Masking objects: None
Two-class or three-class thresholding? : Three classes
Minimize the weighted variance or the entropy? : Weighted variance
Assign pixels in the middle intensity class to the foreground or the background? : Background
Method to calculate adaptive window size: Image size
Size of adaptive window: 10
Use default parameters? : Default
Lower outlier fraction: 0.05
Upper outlier fraction: 0.05
Averaging method: Mean
Variance method: Standard deviation
# of deviations: 2.0

<u>Module 7:</u> Expand Or Shrink Objects:

Select the input objects: Well_FITC_small
Name the output objects: Well_FITC

Select the operation: Shrink objects by a specified number of pixels
Number of pixels by which to expand or shrink: 5
Fill holes in objects so that all objects shrink to a single point? : No
Retain the outlines of the identified objects? : No
Name the outline image: Shrunken Nuclei Outlines

<u>Module 8:</u> Image Math:
Operation: Invert
Raise the power of the result by: 1.0
Multiply the result by: 1.0
Add to result: 0.0
Set values less than 0 equal to 0? : Yes
Set values greater than 1 equal to 1? : Yes
Ignore the image masks? : No
Name the output image: InvertedBF
Image or measurement? : Image
Select the first image: Brightfield
Multiply the first image by: 1.0
Measurement:
Image or measurement? : Image
Select the second image:
Multiply the second image by: 1.0
Measurement:

<u>Module 9:</u> Morph:
Select the input image: InvertedBF
Name the output image: BrightfieldBackground
Select the operation to perform: open
Number of times to repeat operation: Once
Repetition number: 2
Diameter: 31.0
Structuring element: Disk
X offset: 1.0
Y offset: 1.0
Angle: 0.0
Width: 3.0
Height: 3.0
Custom: 5,5,1111111111111111111111111
Rescale values from 0 to 1? : Yes

<u>Module 10:</u> Image Math:
Operation: Subtract
Raise the power of the result by: 1.0
Multiply the result by: 1.0
Add to result: 0.0
Set values less than 0 equal to 0? : Yes
Set values greater than 1 equal to 1? : Yes
Ignore the image masks? : No
Name the output image: BF_worm_image

Image or measurement? : Image
Select the first image: InvertedBF
Multiply the first image by: 1.0
Measurement:
Image or measurement? : Image
Select the second image: BrightfieldBackground
Multiply the second image by: 1.0
Measurement:

<u>Module 11:</u> Mask Image:
Select the input image: BF_worm_image
Name the output image: Masked_BF_worm_image
Use objects or an image as a mask? : Objects
Select object for mask: Well_FITC
Select image for mask: None
Invert the mask? : No

<u>Module 12:</u> Identify Primary Objects:
Select the input image: Masked_BF_worm_image
Name the primary objects to be identified: Worm_objects
Typical diameter of objects, in pixel units (Min, Max): 30, 400
Discard objects outside the diameter range? : Yes
Try to merge too small objects with nearby larger objects? : No
Discard objects touching the border of the image? : No
Method to distinguish clumped objects: Intensity
Method to draw dividing lines between clumped objects: None
Size of smoothing filter: 10
Suppress local maxima that are closer than this minimum allowed distance: 7.0
Speed up by using lower-resolution image to find local maxima? : Yes
Name the outline image: PrimaryOutlines
Fill holes in identified objects? : Never
Automatically calculate size of smoothing filter for declumping? : Yes
Automatically calculate minimum allowed distance between local maxima? : Yes
Retain outlines of the identified objects? : No
Automatically calculate the threshold using the Otsu method? : Yes
Enter Laplacian of Gaussian threshold: 0.5
Automatically calculate the size of objects for the Laplacian of Gaussian filter? : Yes
Enter LoG filter diameter: 5.0
Handling of objects if excessive number of objects identified: Continue
Maximum number of objects: 500
Threshold setting version: 1
Threshold strategy: Automatic
Thresholding method: Otsu
Select the smoothing method for thresholding: Automatic
Threshold smoothing scale: 1.0
Threshold correction factor: 1.0
Lower and upper bounds on threshold: 0.0, 1.0

Approximate fraction of image covered by objects? : 0.01
Manual threshold: 0.0
Select the measurement to threshold with: None
Select binary image: None
Masking objects: None
Two-class or three-class thresholding? : Two classes
Minimize the weighted variance or the entropy? : Weighted variance
Assign pixels in the middle intensity class to the foreground or the background? : Foreground
Method to calculate adaptive window size: Image size
Size of adaptive window: 10
Use default parameters? : Default
Lower outlier fraction: 0.05
Upper outlier fraction: 0.05
Averaging method: Mean
Variance method: Standard deviation
# of deviations: 2.0

Module 13: Measure Object Size Shape:
Select objects to measure: Worm_objects
Calculate the Zernike features? : No

Module 14: Filter Objects:
Name the output objects: Worms
Select the object to filter: Worm_objects
Select the filtering mode: Measurements
Select the filtering method: Limits
Select the objects that contain the filtered objects: None
Retain outlines of the identified objects? : No
Name the outline image: FilteredObjects
Rules file location: Elsewhere...\x7C
Rules file name: rules.txt
Class number: 1
Measurement count: 2
Additional object count: 0
Assign overlapping child to: Both parents
Select the measurement to filter by: AreaShape_Solidity
Filter using a minimum measurement value? : No
Minimum value: 0.7
Filter using a maximum measurement value? : Yes
Maximum value: 0.9
Select the measurement to filter by: AreaShape_MajorAxisLength
Filter using a minimum measurement value? : No
Minimum value: 0.0
Filter using a maximum measurement value? : Yes
Maximum value: 450

Module 15: Identify Secondary Objects:
Select the input objects: Worms

Name the objects to be identified: DilatedWorms
Select the method to identify the secondary objects: Distance - N
Select the input image: Brightfield
Number of pixels by which to expand the primary objects: 5
Regularization factor: 0.05
Name the outline image: SecondaryOutlines
Retain outlines of the identified secondary objects? : No
Discard secondary objects touching the border of the image? : No
Discard the associated primary objects? : No
Name the new primary objects: FilteredNuclei
Retain outlines of the new primary objects? : No
Name the new primary object outlines: FilteredNucleiOutlines
Fill holes in identified objects? : No
Threshold setting version: 1
Threshold strategy: Automatic
Thresholding method: Otsu
Select the smoothing method for thresholding: No smoothing
Threshold smoothing scale: 1.0
Threshold correction factor: 1.0
Lower and upper bounds on threshold: 0.0,1.0
Approximate fraction of image covered by objects? : 0.01
Manual threshold: 0.0
Select the measurement to threshold with: None
Select binary image: None
Masking objects: None
Two-class or three-class thresholding? : Two classes
Minimize the weighted variance or the entropy? : Weighted variance
Assign pixels in the middle intensity class to the foreground or the background? : Foreground
Method to calculate adaptive window size: Image size
Size of adaptive window: 10
Use default parameters? : Default
Lower outlier fraction: 0.05
Upper outlier fraction: 0.05
Averaging method: Mean
Variance method: Standard deviation
# of deviations: 2.0

<u>Module 16:</u> IdentifyTertiaryObjects:
Select the larger identified objects: DilatedWorms
Select the smaller identified objects: Worms
Name the tertiary objects to be identified: WormBackground
Name the outline image: CytoplasmOutlines
Retain outlines of the tertiary objects? : No
Shrink smaller object prior to subtraction? : No

<u>Module 17:</u> Mask Image:
Select the input image: FITC
Name the output image: MaskedFITC

Use objects or an image as a mask? : Objects
Select object for mask: Well_FITC
Select image for mask: None
Invert the mask? : No

<u>Module 18:</u> Correct Illumination Calculate:
Select the input image: MaskedFITC
Name the output image: IllumFITC
Select how the illumination function is calculated: Regular
Dilate objects in the final averaged image? : No
Dilation radius: 1
Block size: 60
Rescale the illumination function? : No
Calculate function for each image individually, or based on all images? : Each
Smoothing method: Fit Polynomial
Method to calculate smoothing filter size: Automatic
Approximate object size: 10
Smoothing filter size: 10
Retain the averaged image? : No
Name the averaged image: IllumBlueAvg
Retain the dilated image? : No
Name the dilated image: IllumBlueDilated
Automatically calculate spline parameters? : Yes
Background mode: auto
Number of spline points: 5
Background threshold: 2.0
Image resampling factor: 2.0
Maximum number of iterations: 40
Residual value for convergence: 0.001

<u>Module 19:</u> Correct Illumination Apply:
Select the input image: MaskedFITC
Name the output image: CorrFITC
Select the illumination function: IllumFITC
Select how the illumination function is applied: Subtract

<u>Module 20:</u> Identify Primary Objects:
Select the input image: CorrFITC
Name the primary objects to be identified: Worm_heads
Typical diameter of objects, in pixel units (Min, Max): 5, 10
Discard objects outside the diameter range? : Yes
Try to merge too small objects with nearby larger objects? : No
Discard objects touching the border of the image? : Yes
Method to distinguish clumped objects: Intensity
Method to draw dividing lines between clumped objects: None
Size of smoothing filter: 10
Suppress local maxima that are closer than this minimum allowed distance: 7.0
Speed up by using lower-resolution image to find local maxima? : Yes
Name the outline image: PrimaryOutlines

Fill holes in identified objects? : Never
Automatically calculate size of smoothing filter for declumping? : Yes
Automatically calculate minimum allowed distance between local maxima? : Yes
Retain outlines of the identified objects? : No
Automatically calculate the threshold using the Otsu method? : Yes
Enter Laplacian of Gaussian threshold: 0.5
Automatically calculate the size of objects for the Laplacian of Gaussian filter? : Yes
Enter LoG filter diameter: 5.0
Handling of objects if excessive number of objects identified: Continue
Maximum number of objects: 500
Threshold setting version: 1
Threshold strategy: Global
Thresholding method: Otsu
Select the smoothing method for thresholding: Automatic
Threshold smoothing scale: 1.0
Threshold correction factor: 1.0
Lower and upper bounds on threshold: 0.004,1.0
Approximate fraction of image covered by objects? : 0.01
Manual threshold: 0.0
Select the measurement to threshold with: None
Select binary image: None
Masking objects: None
Two-class or three-class thresholding? : Two classes
Minimize the weighted variance or the entropy? : Weighted variance
Assign pixels in the middle intensity class to the foreground or the background? : Background
Method to calculate adaptive window size: Image size
Size of adaptive window: 10
Use default parameters? : Default
Lower outlier fraction: 0.05
Upper outlier fraction: 0.05
Averaging method: Mean
Variance method: Standard deviation
# of deviations: 2.0

Module 21: Overlay Outlines:
Display outlines on a blank image? : No
Select image on which to display outlines: Brightfield
Name the output image: OrigOverlay
Outline display mode: Color
Select method to determine brightness of outlines: Max of image
Width of outlines: 1
Select outlines to display: None
Select outline color: cyan
Load outlines from an image or objects? : Objects
Select objects to display: Worms
Select outlines to display: None

Select outline color: yellow
Load outlines from an image or objects? : Objects
Select objects to display: Worm_heads
Select outlines to display: None
Select outline color: Blue
Load outlines from an image or objects? : Objects
Select objects to display: WormBackground

Module 22: Display Data On Image:
Display object or image measurements? : Object
Select the input objects: Worms
Measurement to display: Number_Object_Number
Select the image on which to display the measurements: OrigOverlay
Text color: red
Name the output image that has the measurements displayed: DisplayImage
Font size (points): 12
Number of decimals: 0
Image elements to save: Image
Annotation offset (in pixels): 0
Display mode: Text
Color map: Default
Display background image? : Yes
Color map scale: Use this image\'s measurement range
Color map range: 0.0,1.0

Module 23: Save Images:
Select the type of image to save: Image
Select the image to save: DisplayImage
Select the objects to save: None
Select the module display window to save: None
Select method for constructing file names: From image filename
Select image name for file prefix: Brightfield
Enter single file name: OrigBlue
Number of digits: 4
Append a suffix to the image file name? : Yes
Text to append to the image name: _outlines
Saved file format: png
Output file location: Default Output Folder\x7C
Image bit depth: 8-bit integer
Overwrite existing files without warning? : No
When to save: Every cycle
Rescale the images? : No
Save as grayscale or color image? : Grayscale
Select colormap: Default
Record the file and path information to the saved image? : No
Create subfolders in the output folder? : No
Base image folder: Elsewhere...\x7C
Saved movie format: avi

Module 24: **Relate Objects**

Select the input child objects: Worm_heads
Select the input parent objects: Worms
Calculate child-parent distances? : None
Calculate per-parent means for all child measurements? : No
Calculate distances to other parents? : No
Parent name: None

Module 25: **Measure Object Intensity:**

Hidden: 2
Select an image to measure: FITC
Select an image to measure: Cy3
Select objects to measure: Worms
Select objects to measure: WormBackground

Module 26: **Measure Object Size Shape:**

Select objects to measure: Worms
Calculate the Zernike features? : Yes

Module 27: **Export To Spread sheet:**

Select the column delimiter: Comma (",")
Add image metadata columns to your object data file? : No
Limit output to a size that is allowed in Excel? : No
Select the measurements to export: Yes
Calculate the per-image mean values for object measurements? : No
Calculate the per-image median values for object measurements? : No
Calculate the per-image standard deviation values for object measurements? : No
Output file location: Default Output Folder\x7C
Create a GenePattern GCT file? : No
Select source of sample row name: Metadata
Select the image to use as the identifier: None
Select the metadata to use as the identifier: None
Export all measurement types? : Yes
:
Image\x7CFileName_Brightfield,Worms\x7CIntensity_MinIntensity_Cy3,Worms\x7CIntensity_MinIntensity_FITC,Worms\x7CIntensity_IntegratedIntensityEdge_FITC,Worms\x7CIntensity_IntegratedIntensityEdge_Cy3,Worms\x7CIntensity_StdIntensity_Cy3,Worms\x7CIntensity_StdIntensity_FITC,Worms\x7CIntensity_MassDisplacement_Cy3,Worms\x7CIntensity_MassDisplacement_FITC,Worms\x7CIntensity_UpperQuartileIntensity_Cy3,Worms\x7CIntensity_UpperQuartileIntensity_FITC,Worms\x7CIntensity_IntegratedIntensity_FITC,Worms\x7CIntensity_IntegratedIntensity_Cy3,Worms\x7CIntensity_MinIntensityEdge_FITC,Worms\x7CIntensity_MinIntensityEdge_Cy3,Worms\x7CIntensity_MADIntensity_Cy3,Worms\x7CIntensity_MADIntensity_FITC,Worms\x7CIntensity_MeanIntensity_Cy3,Worms\x7CIntensity_MeanIntensity_FITC,Worms\x7CIntensity_MeanIntensityEdge_FITC,Worms\x7CIntensity_MeanIntensityEdge_Cy3,Worms\x7CIntensity_MaxIntensity_Cy3,Worms\x7CIntensity_MaxIntensity_FITC,Worms\x7CIntensity_MedianIntensity_FITC,Worms\x7CIntensity_MedianIntensity_Cy3,Worms\x7CIntensit

y_LowerQuartileIntensity_Cy3,Worms\x7CIntensity_LowerQuartileIntensity_FITC,Worms\x7CIntensity_MaxIntensityEdge_FITC,Worms\x7CIntensity_MaxIntensityEdge_Cy3,Worms\x7CIntensity_StdIntensityEdge_Cy3,Worms\x7CIntensity_StdIntensityEdge_FITC,Worms\x7CAreaShape_Perimeter,Worms\x7CAreaShape_MinorAxisLength,Worms\x7CAreaShape_Center_Y,Worms\x7CAreaShape_Center_X,Worms\x7CAreaShape_Area,Worms\x7CAreaShape_MinFeretDiameter,Worms\x7CAreaShape_Solidity,Worms\x7CAreaShape_MaxFeretDiameter,Worms\x7CAreaShape_MeanRadius,Worms\x7CAreaShape_EulerNumber,Worms\x7CAreaShape_MedianRadius,Worms\x7CAreaShape_Compactness,Worms\x7CAreaShape_Extent,Worms\x7CAreaShape_Eccentricity,Worms\x7CAreaShape_MaximumRadius,Worms\x7CAreaShape_FormFactor,Worms\x7CAreaShape_Zernike_1_1,Worms\x7CAreaShape_Zernike_0_0,Worms\x7CAreaShape_Zernike_3_1,Worms\x7CAreaShape_Zernike_3_3,Worms\x7CAreaShape_Zernike_2_2,Worms\x7CAreaShape_Zernike_2_0,Worms\x7CAreaShape_Zernike_5_1,Worms\x7CAreaShape_Zernike_5_3,Worms\x7CAreaShape_Zernike_5_5,Worms\x7CAreaShape_Zernike_4_2,Worms\x7CAreaShape_Zernike_4_4,Worms\x7CAreaShape_Zernike_4_0,Worms\x7CAreaShape_Zernike_7_1,Worms\x7CAreaShape_Zernike_7_3,Worms\x7CAreaShape_Zernike_7_5,Worms\x7CAreaShape_Zernike_7_7,Worms\x7CAreaShape_Zernike_6_2,Worms\x7CAreaShape_Zernike_6_0,Worms\x7CAreaShape_Zernike_6_4,Worms\x7CAreaShape_Zernike_6_6,Worms\x7CAreaShape_Zernike_9_1,Worms\x7CAreaShape_Zernike_9_3,Worms\x7CAreaShape_Zernike_9_5,Worms\x7CAreaShape_Zernike_9_7,Worms\x7CAreaShape_Zernike_9_9,Worms\x7CAreaShape_Zernike_8_0,Worms\x7CAreaShape_Zernike_8_2,Worms\x7CAreaShape_Zernike_8_4,Worms\x7CAreaShape_Zernike_8_6,Worms\x7CAreaShape_Zernike_8_8,Worms\x7CAreaShape_MajorAxisLength,Worms\x7CAreaShape_Orientation,Worms\x7CChildren_Worm_heads_Count

Representation of Nan/Inf: NaN
Add a prefix to file names? : Yes
Filename prefix: Worm_Measurements
Overwrite existing files without warning? : No
Data to export: Do not use
Combine these object measurements with those of the previous object? : No
File name: DATA.csv
Use the object name for the file name? : Yes
